# ApoA1-driven cholesterol efflux and macrophage polarization orchestrate T-cell differentiation towards controlling *Leishmania donovani* pathogenesis

**DOI:** 10.1038/s41598-025-30130-1

**Published:** 2025-12-03

**Authors:** Vikash Kumar, Dayakar Alti, Shobha Kumari, Ravi Ranjan, Divya Prasad, Veer Singh, Abhik Sen, Pradeep Das, Krishna Pandey, Ashish Kumar

**Affiliations:** 1 Department of Biochemistry, ICMR-Rajendra Memorial Research Institute of Medical Science, Patna, 800007 India; 2 Department of Immunology, ICMR-Rajendra Memorial Research Institute of Medical Science, Agamkuan, Patna, 800007 India; 3 Aquatic Toxicology Laboratory, Department of Zoology, Patna University, Patna, 800005 India; 4 Department of Molecular Biology, ICMR-Rajendra Memorial Research Institute of Medical Science, Agamkuan, Patna, 800007 India; 5 ICMR-National Institute for Research in Bacterial Infections, Kolkata, 700010 India; 6 Department of Clinical Medicine, ICMR-Rajendra Memorial Research Institute of Medical Science, Agamkuan, Patna, 800007 India

**Keywords:** *Leishmania donovani*, Apolipoprotein A1, Cholesterol efflux, M1-macrophages, Human PBMCs, Th1-polarization, Cell biology, Diseases, Immunology

## Abstract

Lipid metabolism plays a decisive role in host-pathogen interactions and immune regulation, with apolipoproteins (Apo) being central to this process. However, their role in leishmaniasis remains unexplored. Herein, we deliberate the immunoregulatory function of ApoA1 during *Leishmania donovani* infection using THP-1-derived macrophages alone and in combination with T lymphocytes derived from human PBMC. We found low serum ApoA1 levels in active VL and PKDL than in healthy controls. It was shown that direct interaction of ApoA1 with ABCA1 (ATP-binding cassette transporter A1) on macrophages promotes cholesterol efflux, reflected by increased HDL levels and reduced total cellular cholesterol. This phenomenon was associated with reduced *Leishmania* infectivity and its downstream signaling in macrophages, i.e., downregulation of PPAR-γ and the endoplasmic reticulum-stress marker CHOP. Additionally, ApoA1 in the presence of extracellular HDL slightly promoted macrophage polarization towards M1, as indicated by increased expression of IL-12 and iNOS2 or nitric oxide production, alongside reduced expression of M2 phenotype-associated markers, including IL-10 and arginase. In co-culture with PBMC-derived T-cells, ApoA1-primed macrophages facilitated Th1 polarization, as demonstrated by increased IFN-γ and STAT1, and indirectly by reduced expression of Th2-specific markers (GATA-3 and IL-4). Overall, these results implicate ApoA1 as a vital immunomodulatory factor and potential therapeutic target in leishmaniasis.

**Supplementary Information:**

The online version contains supplementary material available at 10.1038/s41598-025-30130-1.

## Introduction

Visceral leishmaniasis (VL) is known to be a zoonotic vector-mediated illness, chiefly attributable to parasites of the genus *Leishmania*, particularly *L. donovani* and *L. infantum*. Within South Asia’s Indian subcontinent, transmission of VL primarily occurs via hematophagous bites of infected female Phlebotomus sandflies. This pathological condition predominantly targets the reticuloendothelial system, adversely impairing pivotal organs including the spleen, liver, and bone marrow. These organs perform a central role in immune defence in the host, and their dysfunction contributes significantly to the pathogenesis of VL^1^. During the pathophysiology of leishmaniasis, innate as well as adaptive immune mechanisms work in concert to combat the parasite. Macrophages (MΦ) are front-line warriors of innate immunity, serving as the primary effector cells in host defence. In contrast, the adaptive immune response is driven by T and B lymphocytes, which generate specific antibodies and cytokines essential for parasite clearance^2,3^. This intricate synergy between innate immune response and adaptive cellular immunity is crucial for effective *Leishmania* elimination and the prevention of disease progression. As MΦ are primary targets of *Leishmania* infection, which can determine the fate of the disease^4^, understanding their role for extracellular stimuli is more critical. In general, MΦ are categorized into two subsets: M1 and M2. M1 phenotype exhibits pro-inflammatory response and enhances pathogen clearance through robust antimicrobial activity, while M2 phenotype possesses anti-inflammatory properties that can support parasite persistence and survival^3,5,6^. Understanding the immunomodulatory dynamics between the MΦ subsets is essential for developing targeted interventions against *Leishmania* infections.

Beyond their canonical role in maintaining host cell membrane structural integrity, lipids actively participate in modulating host-pathogen interactions during *Leishmania* infection. They significantly influence disease progression by regulating parasite entry, antigen presentation, immune modulation, drug responsiveness, and energy homeostasis^7^. Individuals with active VL exhibit pronounced alterations in serum lipid profiles, marked by diminished Apolipoprotein A1 (ApoA1), cholesterol, high-density lipoprotein (HDL), and low-density lipoprotein (LDL) levels, coupled with elevated triglycerides and very low-density lipoprotein (VLDL) compared to healthy controls^7–10^. The precise mechanisms underlying the depletion of ApoA1 and cholesterol in VL patients remain unclear. However, a plausible hypothesis suggests that hepatic dysfunction induced by *Leishmania* infection may be responsible, assuming that the liver serves as the principal site for the biosynthesis of ApoA1 and cholesterol.

ApoA1, the major protein constituent responsible for HDL structure and function, is predominantly produced in the liver, although it is also produced in smaller amounts in the small intestine. It plays a pivotal function in maintaining lipid equilibrium by facilitating RCT (reverse cholesterol transport), wherein surplus cholesterol is extracted from peripheral tissues, including MΦ, and transported back to the hepatic system for subsequent clearance from the body^11,12^. A key component of this pathway is the interaction between ApoA1 and ABCA1, a membrane-associated lipid transporter that arbitrates the efflux of cellular cholesterols and phospholipids to ApoA1. This interaction promotes the formation of nascent HDL particles, thereby serving as a critical regulatory mechanism for maintaining cellular cholesterol homeostasis^13–15^. Cholesterol-rich membrane microdomains critically mediate the efficient presentation of Leishmania-derived antigens by infected MΦ to T and B lymphocytes, a process essential for the activation of adaptive immune responses^16,17^. Given the integral role of cholesterol in both parasite entry and immune surveillance, its regulation within host cells holds significant immunopathological relevance. Furthermore, ApoA1 has gained attention for its immunomodulatory effects on MΦ in various diseases, including atherosclerosis, diabetes, and other inflammatory disorders^18–20^. However, its role in leishmaniasis remains largely unexplored. This research endeavored to investigate the immunoregulatory functions of ApoA1 in experimental VL using human MΦ cell lines and peripheral blood mononucleated cells (PBMC) models, potentially highlighting its therapeutic relevance.

## Materials and methods

### Ethical consent and collection of samples

This investigation was executed following the sanction of the institutional ethics committee, ICMR-Rajendra Memorial Research Institute of Medical Sciences, Agamkuan, Patna (Reference No.: RMRI/EC/24/2021), certifying strict adherence to all relevant ethical guidelines. Following the acquisition of informed consent, we recruited three distinct groups: healthy controls, active VL patients, and post-kala-azar dermal leishmaniasis (PKDL) patients, each group comprising 30 participants. All participants were aged 18 years or older and randomly included both sexes. Active VL and PKDL patients were commonly characterized based on rK39 (recombinant kinesin antigen of 39 residues) reactivity and parasite positivity, and typically characterized based on clinical history (e.g., fever and splenomegaly in VL; macular, papular, or nodular skin lesions in PKDL). Healthy controls were defined as individuals who tested negative for rK39 and had no history of VL/PKDL. Individuals exhibiting concurrent infections of HIV and VL were rigorously excluded from this study. To obtain the serum from VL and PKDL patients, we meticulously drew 2 mL of blood from each participant. In contrast, 10 mL of blood was obtained from healthy donors for both serum separation as well as PBMC isolation. These samples were subsequently used for further experimental analyses. Clinical isolates of *Leishmania* were acquired from splenic aspirates of VL patients. The aspirates were processed and incubated in M199 medium (Gibco), meticulously calibrated to a pH of 7.4 and a consistent temperature of 24 °C. Additionally, this medium contains 20% FBS (fetal bovine serum: heat-inactivated) from Gibco and 1% penicillin-streptomycin solution from Sigma-Aldrich. Amastigotes residing in splenic aspiration were slowly transformed into flagellated promastigotes and subsequently continued in the M199 medium supplemented with 10% FBS. Prior to the subculture, the isolates were characterized by the PCR (polymerase chain reaction) amplification of kinetoplast DNA (kDNA) using *L. donovani-*specific primers (forward: 5′-CTTTTCTGGTCCTCCGGGTAGG—3′ and reverse 5′—CACCCGGCCCTATTTTACACCAA—3′).

### Estimation of serum ApoA1 levels

Serum ApoA1 concentration of healthy individuals, active VL, and PKDL patients was measured by the Human Apolipoprotein A1 ELISA (enzyme-linked immunosorbent assay) Kit (Invitrogen) method. Firstly, wells were loaded with serially diluted standards and serum samples (100 µL each), and allowed for incubation at RT (room temperature) for 2.5 h with gentle stirring. After that, each well was emptied and rinsed four times utilizing a 1X Wash Buffer (300 µL). Next, in each well, 100 µL of biotin conjugate was introduced and allowed to incubate for 1 h at RT. The wells were then rigorously rinsed once again, after which HRP enzyme (Horseradish Peroxidase) was applied and allowed to incubate at RT for 45 min. Following repeated washing, each well received ~ 100 µL of TMB substrate, with incubation proceeding for 30 min in the dark to develop a blue color. The enzymatic activity was then halted by the application of 50 µL of stop solution, followed by instant optical density measurement at λ = 450 nm in an ELISA microplate reader (Bio-Rad). Quantification of serum ApoA1 was achieved through interpolation based on a standardized calibration curve.

### THP-1 cell culture and its differentiation into MΦ

As a part of this study, THP-1 monocytic cells have been sourced from NCCS (National Centre for Cell Science), Pune, India. They have been maintained in sterile 25 cm^2^ vented flasks with RPMI-1640 complete medium (Gibco) enriched with 1% penicillin-streptomycin solution and 10% FBS under standard conditions of 37 °C and 5% CO_2_ atmosphere with humidity. To ensure optimal growth and viability, the culture medium was replenished every 3–4 days.In order to induce differentiation, we plated 1 × 10^6^ monocytes per well in a 6-well plate with 20 nM PMA (phorbol 12-myristate 13-acetate; Sigma-Aldrich) and followed this up by 48 h of incubation. Following incubation, the plate was thoroughly rinsed usingRPMI-1640incomplete (FBS-free) medium and subsequently maintained in fresh complete medium for 24 h to attain full differentiation of adherent MΦ.

### ApoA1-ABCA1 interaction in MΦ—confocal microscopy

To examine the likely interaction of ApoA1 with ABCA1 in THP-1-derived MΦ, differentiated sterile coverslips in 6-well plates and incubated them with recombinant human ApoA1 (10 µg/mL; Sigma-Aldrich) for a period of 6 h. Incubation was followed by 4% paraformaldehyde fixation for 10 min, permeabilization for 5 min with 1% Triton X-100 and blocking for 2 h with 3% BSA (bovine serum albumin). For the immunofluorescence assay, the cells underwent staining with FITC (fluorescein isothiocyanate)-labeled anti-ABCA1 (1:250 dilution) and PE (phycoerythrin)-labeled anti-ApoA1 (1:250 dilution) antibodies (Santa Cruz Biotechnology) for 1 h at RT in the dark. After three rinses with 1X PBS, the coverslips were fixed onto glass slides utilizing a DPX mounting medium (Sigma-Aldrich). Then, a confocal microscope (Zeiss LSM 880) was employed to capture images for the study of protein-protein interactions.

### ApoA1-mediated cholesterol efflux assay

#### Lipid extraction from MΦ

To evaluate ApoA1-mediated cholesterol efflux from MΦ, cells were subjected to stimulation with 10 µg/mL of ApoA1 for a duration of 24 h. After incubation, cells and supernatant medium were collected for total lipid extraction using the method described by Paulazo and Sodero^21^. Following PBS wash, lipid extraction was performed as below: firstly, cells were subjected to a 1 mL treatment of a chloroform-methanol mixture (2:1 ratio) and subjected to vortexing, six pulses and each lasting for 20 s with 5 min intervals. Secondly, a mixture of chloroform (166.6 µL) and water (300 µL) was added, vortexed, and centrifugation at 1300×*g* at RT for 10 min. The top aqueous phase was aspirated, and the residual organic phase was subjected to a repeated extraction as outlined in step 2. Finally, the organic phase was evaporated at 40 °C using a SpeedVac vacuum concentrator. The resulting lipid extract was reconstituted in 1 mL of isopropanol and kept at − 20 °C until use.

#### Estimation of cellular cholesterol levels in MΦ

The total cholesterol content in the above reconstituted samples was analyzed using reverse-phase-HPLC (Shimadzu LC-2010 A system), provided with a Kinetex 2.6 μm C18 100 Å column. Using a mobile phase composed of isopropanol, acetonitrile, and water (60:30:10), chromatographic separation was achieved at a controlled flow rate of 1.0 mL/min. The optimal temperature of the column was regulated at 28 °C, with detection carried out at an ideal λ of 205 nm. Cholesterol detection was made by its full-length ultraviolet (UV) absorption spectrum and retention time (11 min). Quantification was performed by integrating the area under the corresponding chromatographic peak. In order to prepare working standards, 25 mg/mL cholesterol stock solutions from Sigma-Aldrich were serially diluted in isopropanol from 100 to 3.125 µg/mL. Each standard was analyzed in quadruplicate to ensure robustness and reliability, and the resulting peak areas were averaged to obtain precise cholesterol concentrations.

#### Estimation of extracellular HDL

To evaluate the role of ApoA1 in cholesterol efflux from MΦ, we measured HDL levels in previously stored culture supernatants (referencing "Lipid extraction from MΦ" Section) using the HDL-cholesterol kit (PRIMAN^R^Homogenous Method). Briefly, 150 µL of Reagent 1 (consisting of Good’s buffer at 100 mmol/L, N-(2-Hydroxy-3-sulfopropyl)-3,5-dimethoxyaniline sodium salt (HDAOS) at 0.42 mmol/L, Catalase at 600 KU/L, Cholesterol oxidase at 380 U/L and Cholesterol esterase at 600 U/L) was added in 2 µL of culture sample incubated for 15–20 min at RT to consume non-HDL free cholesterol and to produce colorless reactant. Then, 50 µL of Reagent 2 (consisting of Good’s buffer at 100 mmol/L, 4-aminoantipyrine at 1.0 mmol/L, Peroxidase > 2.8 U/mL, and Surfactant < 2%) was transferred to the above reactant and allowed for incubation at RT for 20 min, which results in HDL solubilization due to surfactant action. Cholesterol esterase and cholesterol oxidase enzymes then metabolize the HDL, which leads to the production of H_2_O_2_, which is again metabolized by the peroxidase enzyme in the vicinity of 4-aminoantipyrine and HDAOS. Finally, it results in the production of Quinonimine, a red color product, whose levels are proportional to the HDL levels, and was measured at 600 nm using a fully automated biochemistry analyzer (MindrayBS-240).

### Rate of infectivity in ApoA1-primed MΦ

To investigate the impact of ApoA1-induced cholesterol efflux from MΦ on parasite infectivity and their intracellular growth, we cultured MΦ as described previously ("THP-1cell culture and its differentiation into MΦ" and "ApoA1-ABCA1 interaction in MΦ—Confocal microscopy" Sections) for 24 h. After that, cells were co-cultured with stationary-phase *Ld-*promastigotes for 12 h in a 1:10 ratio in ApoA1-augmentedRPMI-1640 complete medium at 37 °C and a 5% CO_2_ atmosphere. After thorough washing of the plate to discard non-internalized parasites, infected macrophages were maintained for a further 12 h under the same conditions. After ApoA1-priming and *Ld*-infection, MΦ were stained using May-Grünwald Giemsa^22^, and the rate of infectivity was determined by measuring the proportion of infected MΦ and counting intracellular amastigotes/100 MΦ using light microscopy (Nikon Eclipse Ci).

### ApoA1-mediatedMΦ polarization during Ld-infection

The differentiated MΦ were employed in four experimental groups: (1) MΦ without ApoA1 or *Ld*-infection (negative control), (II) MΦ with 100 ng/ml lipopolysaccharide (LPS) stimulation (positive control), (III) MΦ only with *Ld*-infection, and(IV)ApoA1-primed MΦ with *Ld*-infection (as mentioned in "Rate of infectivity in ApoA1-primed MΦ" Section) and maintained in RPMI-1640 complete medium, without phenol-red, for 24 h. After that, the culture supernatants and cells were harvested for subsequent analysis.

#### Western blot analysis of signaling molecules

To explore the downstream signaling molecules response upon MΦ polarization, an immuno-blotting assay was performed. In brief, to initiate the cytosolic proteins extraction, the MΦ were resuspended in radio-immunoprecipitation assay (RIPA) buffer and subjected to five consecutive cycles of freeze-thaw, each consisting of 10 min immersion in liquid nitrogen and 5 min incubation in a water bath adjusted at 37 °C. Following lysis, lysed samples were subsequently centrifuged at 13,000×*g* for 15 min at 4 °C. The supernatants, which contain soluble proteins, were carefully collected for further analysis. Approximately 10 µg protein from each experimental group was resolved under reducing conditions on a 12% SDS-PAGE gel and then subsequently blotted on PVDF (polyvinylidene difluoride) membranes utilizing a semi-dry blotter (Bio-Rad). In order to minimize non-specific antibody interactions with the membranes, the membranes were blocked with 3% BSA in PBST (1X PBS containing 0.05% Tween-20) for 1 h at room temperature after they had been transferred. The blot membranes underwent overnight incubation at 4 °C with mouse anti-human C/EBP homologous protein (CHOP-1) & peroxisome proliferator-activated receptor (PPAR-γ)1° antibodies (1:1000; Invitrogen). Subsequently, blots were rinsed and exposed to AP (alkaline phosphatase)-labeled goat anti-mouse IgG2° antibody (1:2000 dilution) for 1 h at RT. The detection of protein bands was achieved by applying the chromogenic substrate system NBT/BCIP, comprising nitro blue tetrazolium and 5-bromo-4-chloro-3-indolyl phosphate. Images were captured utilizing a gel documentation system (Vilber), and densitometry analysis of the protein bands was achieved with BioVision software.

#### Gene expression analysis of key immuneplayers

To explore the ApoA1 roles in regulating the gene expression of key immune components associated withMΦ polarization, we employed the reverse transcriptase qPCR method. Briefly, the MΦ were applied for total RNA extraction utilizing the Trizol method (Invitrogen). Quality of RNA was assessed using NanoDrop spectrophotometer (Biolinkk). The synthesis of cDNA from total RNA extracts was achieved employing M-MLV Reverse Transcriptase (Promega). Custom-designed primers (IDT) listed in Table 1 were used for target gene amplification utilizing SYBR Green master mix (Roche) in a qPCR system (CFX96 Dx: Bio-Rad). The qPCR reactions (20 µL) contained 0.1 µL cDNA representing 30 ng RNA input. The thermal cycling protocol included 45 cycles comprising 95 °C denaturation for 15 s, annealing at gene-specific temperatures for 30 s, and 72 °C extension for 30 s. Specific amplification was confirmed through melting curve analysis, adhering to the manual. Glyceraldehyde-3-phosphate dehydrogenase (GAPDH) gene was employed as the endogenous control for normalization, while relative gene expression was quantified using CFX Maestro Software.

**Table 1:** Target genes and their specific primers, including melting temperatures for RT-qPCR expression analysis, and the amplicon sizes are tabulated.

S. No.	Gene	Primer sequence (5′ → 3′)		Tm (°C)	Amplicon length (bp)
1	GAPDH	F	CAAGAGCACAAGAGGAAGAGAG	62	102
		R	CTACATGGCAACTGTGAGGAG	62	
2	IL-12 A	F	TGCCTTCACCACTCCCAAAACC	62	100
		R	CAATCTCTTCAGAAGTGCAAGGG	62	
3	IL-10	F	TCTCCGAGATGCCTTCAGCAGA	62	126
		R	TCAGACAAGGCTTGGCAACCCA	62	
4	IL-4	F	CCTCACATTGTCACTGCAAATC	62	124
		R	AGGTGATATCGCACTTGTGTC	62	
5	IFN-ϒ	F	GAGTGTGGAGACCATCAAGGAAG	62.62	124
		R	TGCTTTGCGTTGGACATTCAAGTC	62.79	
6	iNOS2	F	GTCAGAGTCACCATCCTCTTTG	58.41	129
		R	GCAGCTCAGCCTGTACTTATC	58.52	
7	Arginase	F	GGGAAGACACCAGAAGAAGTAA	62	116
		R	GGTGGGTTAAGGTAGTCAATAGG	62	
8	STAT-1	F	GGTTGAACCCTACACGAAGAA	60	200
		R	TAGGGCCATCAAGTTCCATTG	60	
9	GATA-3	F	TTGCATCTGGGTAGCTGTAAG	62	138
		R	TGGCCAGTGAAAGGAAACA	62	

#### Cytokine analysis

To further validate ApoA1-mediated MΦ polarization through cytokine profiling, ELISA was performed using commercially available uncoated ELISA kits (Thermo Fisher Scientific). For assessment of cytokine levels (IL-4, IL-10, IL-12 and IFN-γ), 100 µL aliquots of culture supernatant from each experimental group were processed following the protocols stipulated by the manufacturer. To validate experimental repeatability and accuracy, all assays were conducted in triplicate. The optical density, at λ = 450 nm, of the samples was measured in an ELISA microplate reader (Bio-Rad), and cytokine concentrations were determined based on their respective standard curves.

#### Nitric oxide estimation

To examine the ApoA1 effect on nitric oxide (NO) production from MΦ in response to the *L. donovani* challenge, we adopted a protocol from Kumari et al.^23^ in this regard. Briefly, 100 µL of supernatant from each experimental group was incubated with nitrate reductase (300 U/µL) along with cofactor NADPH (25 µM) for 10 min at RT. This was succeeded by 30-min incubation in Griess reagent, comprising 2% sulfanilamide in 5% phosphoric acid and 0.2% N-(1-naphthyl)ethylenediamine dihydrochloride. The absorbance of the resultant chromophore was measured at λ = 540 nm using an ELISA microplate reader (Bio-Rad). Nitrite levels were measured against a sodium nitrite standard curve, enabling the quantification of NO production and expressed as µM per 10^6^ cells.

### Study of ApoA1-driven MΦ role in T cell polarization

#### PBMC isolation

As part of the study, PBMCs were isolated from healthy donors by using the Ficoll-Paque Plus density gradient centrifugation (Cytiva; density 1.077 g/mL) method. Briefly, 10 mL of whole blood was diluted in a 1:1 ratio with 1X PBS and gently layered over 6 mL of Ficoll-Paque Plus solution preloaded into a 50 mL Falcon tube, followed by centrifugation at 500×*g* for 30 min without applying brakes to allow for optimal layer separation. Following centrifugation, the PBMC-rich buffy coat was carefully harvested, washed thrice in PBS via centrifugation at 350×*g* for 10 min, and then resuspended in isolation buffer for subsequent experimental procedures.

#### T-cell separation from PBMCs

The T-cells were isolated from PBMCs utilizing the Dynabeads™ Untouched™ Human T Cells Kit (Invitrogen), adhering to the manufacturer’s protocol. Briefly, 100 µL of PBMCs, consisting of ~ 1 × 10^8^ cells/mL, was taken into a tube of 5 mL capacity and mixed with 20 µL of FBS and 20 µL of the supplied antibody mix. The cell-antibody mixture was kept for 20 min at 4 °C. Subsequent to incubation, 1 mL isolation buffer was added and mixed gently by tilting the tube several times. We disposed of the supernatant with care and resuspended the pellet in 100 µL of isolation buffer. Subsequently, 100 µL of pre-washed Depletion Dynabeads was mixed with suspension, thoroughly mixed, and set to incubate at RT for 15 min while gently tilting and rotating throughout the process. Following incubation, the tube containing the suspension was supplemented with 1 mL isolation buffer and placed in a magnetic stand for 2 min to facilitate magnetic separation. The supernatant, containing the negatively selected untouched T cells, was then carefully collected into a new tube. To ensure the efficient isolation, we characterised the T-cells, followed by staining with Alexa Fluor™ 647-conjugated anti-human CD3-antibody (Invitrogen)using flow cytometry (BD FACSDiva 8.0.3).

#### MΦand T cell co-culture

Following purification, T cells were used to co-culture with the MΦ in a ratio of 1:2 for 72 h under standard conditions (37 °C, 5% CO_2_). Prior to this, MΦ were stimulated with ApoA1, followed by infection with *Ld* as detailed in "Rate of infectivity in ApoA1-primed MΦ" Section. The non-internalized parasites were thoroughly washed out with incomplete RMPI medium and left for the next 12 h under the same conditions. After repeating the washes, these infected macrophages were co-cultured with T-cells as mentioned above. After co-culturing, the non-adherent T-cell soup was carefully harvested &centrifuged at 700×*g* for 10 min. The supernatant was collected for cytokine quantification (IL-4 and IFN-γ) via ELISA, and the T-cell pellet was used for RNA isolation, followed by RT-qPCR analysis of target gene [signal transducer and activator of transcription (STAT-1) and GATA-3] expression as mentioned in "Cytokine analysis" and "Nitric oxide estimation" Sections.

### Statistical analysis

By using the GraphPad Prism software, version 4.0, the statistical analysis was carried out using one-way ANOVA and unpaired Student’s t-test, with a threshold value of *p* ≤ 0.05 used as the threshold for statistical significance to be established for this study.

## Results

### Active VL/PKDL patients detected with low serum ApoA1 levels

**Fig. 1:**
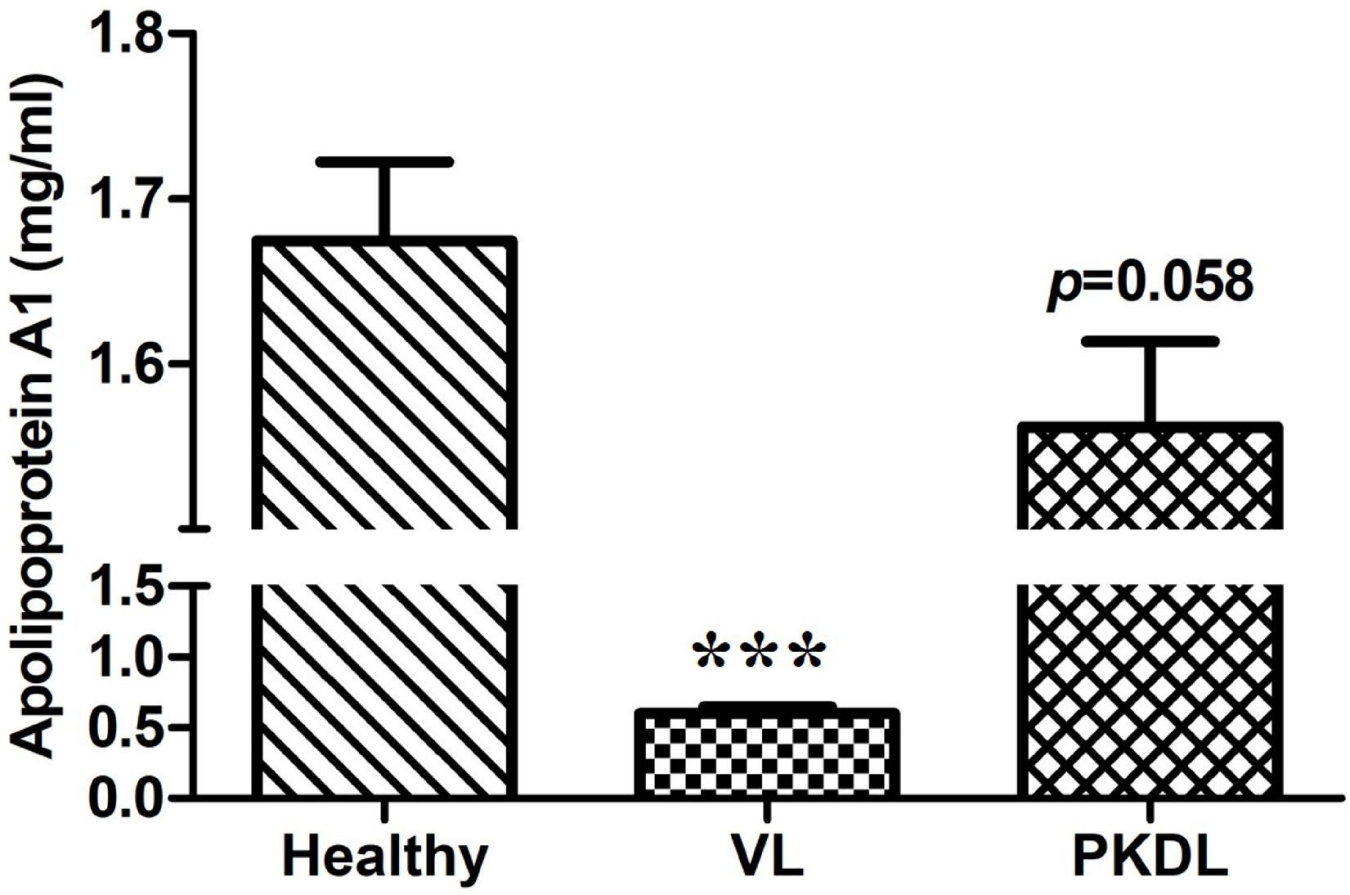
ApoA1 profile in clinical samples. The bar graph shows mean ± SEM of ApoA1 levels (mg/ml) in serum of healthy individuals, active VL, and PKDL patients. Statistical comparisons were performed using one-way ANOVA; significance is denoted by ***p ≤ 0.001.

To understand the fate of ApoA1 in leishmaniasis, as a preliminary assessment, we quantified serum ApoA1 levels from VL and PKDL patients in comparison to healthy individuals. The result shows serum ApoA1 levels were significantly lower in active VL, measuring 0.6019 ± 0.04281 mg/mL (*p* ≤ 0.001), compared to 1.674 ± 0.04797 mg/mL in healthy controls. In contrast, PKDL patients exhibited ApoA1 levels of 1.561 ± 0.05196 mg/mL were not statistically different (*p* = 0.058) from healthy participants (Fig. 1). These observations suggest a strong link between the hepatic origin of ApoA1 and its involvement in the systemic pathogenesis of L. donovani in VL, as opposed to its limited association with the localized dermal manifestations of PKDL.

### ApoA1 interacts with ABCA1 transporter in MΦ

**Fig. 2:**
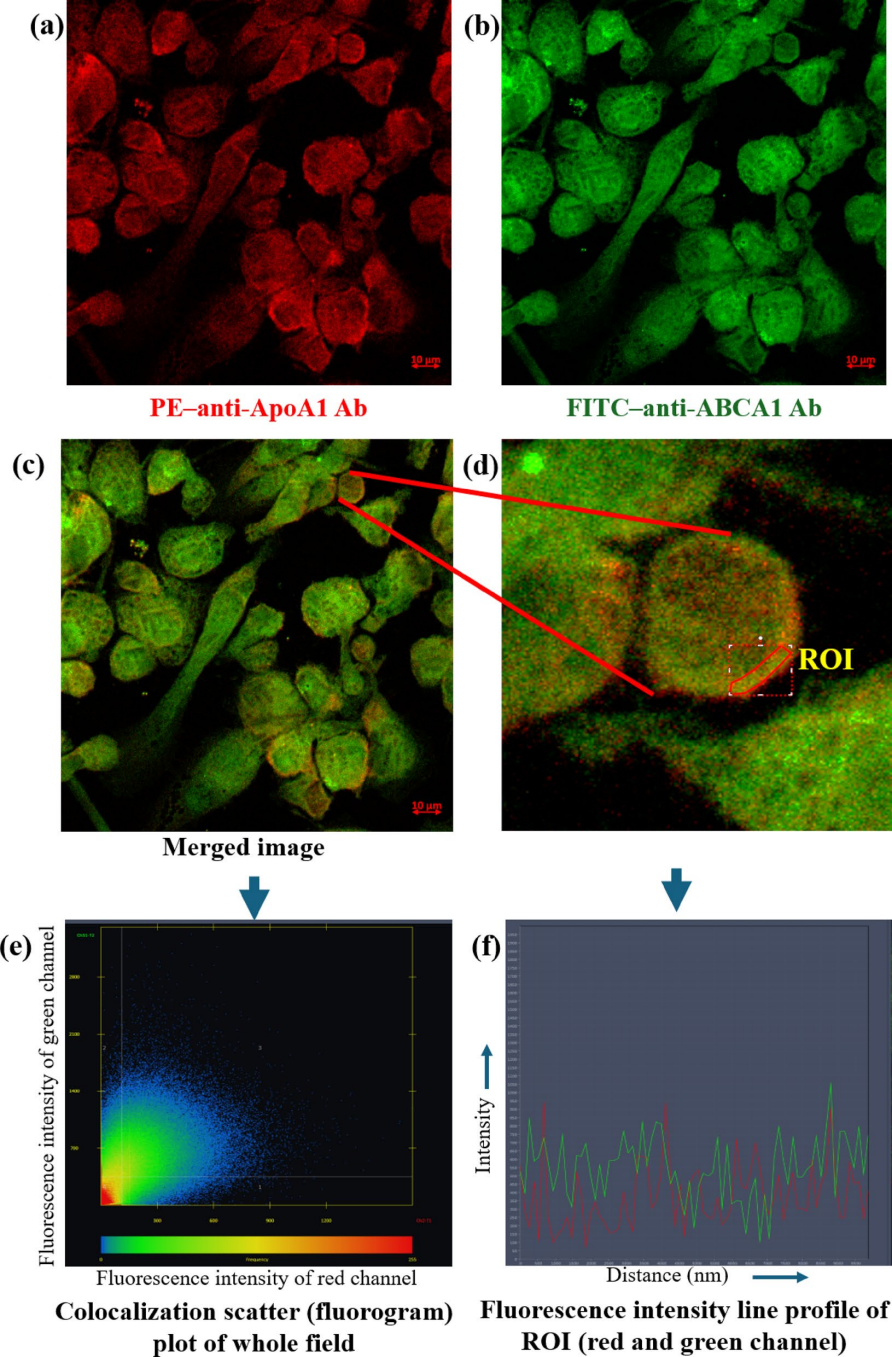
Confocal microscopy of ApoA1-ABCA1 interaction in THP-1 macrophages. The images show (**a**) the localization of ApoA1 with red fluorescence, (**b**) ABCA1 transporter with green fluorescence, (**c**) co-localization of ApoA1 and ABCA1 in yellow fluorescence, (**d**) Region of interest (ROI), (**e**) Colocalization scatter (fluorogram) plot of the whole field, and (**f**) Fluorescence intensity line profile of ROI.

To establish the fact that cholesterol efflux from MΦ, the initial evidence focused on confirming the interaction between ApoA1 and the ABCA1 transporter. Confocal microscopy revealed membrane localization of ApoA1 and ABCA1, marked by red and green fluorescence, respectively (Fig. 2a, b). The merged images displayed distinct yellow fluorescence (Fig. 2c, d), indicating notable co-localization of the two proteins. This spatial overlap was further substantiated through overlay analyses of fluorescence signals (Fig. 2e, f), underscoring the functional interaction. These findings provide compelling evidence of ApoA1 and ABCA1 co-association at the macrophage membrane, supporting their cooperative role in regulating intracellular cholesterol transport during *Leishmania* infection.

### ApoA1 reduces total cellular cholesterol levels

**Fig. 3:**
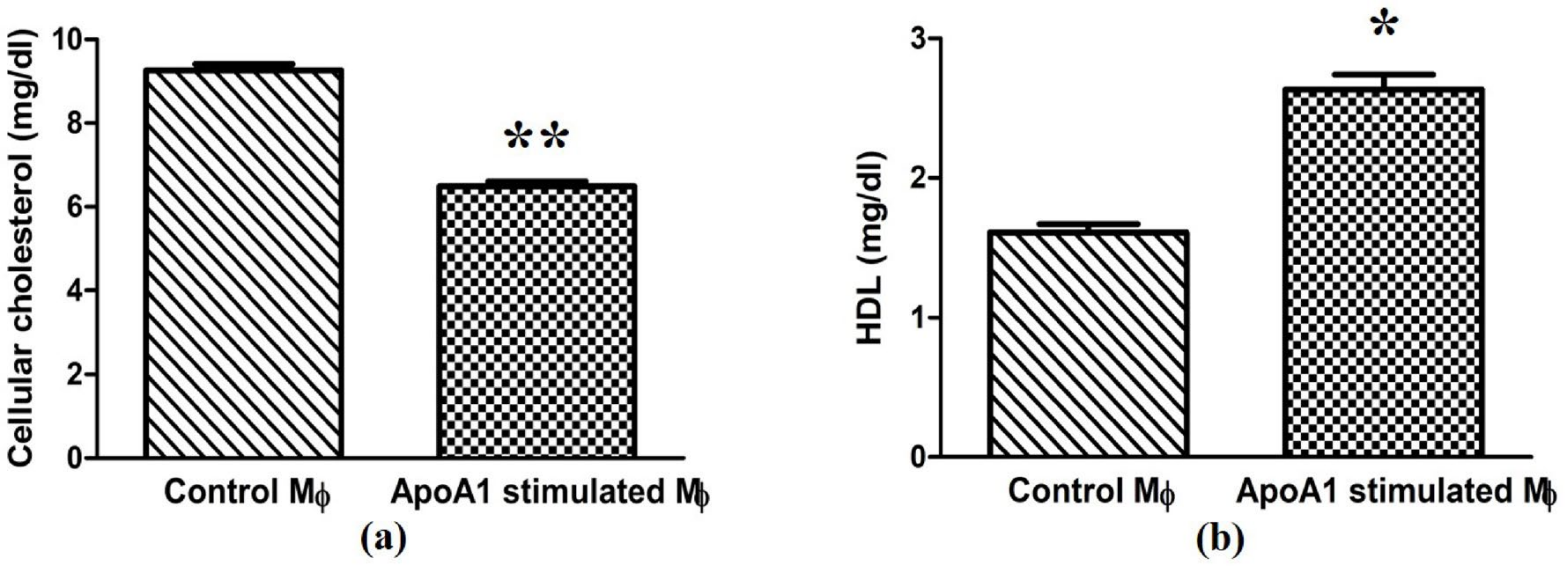
Bar graphs illustrate the impact of ApoA1 on intracellular cholesterol and extracellular HDL concentrations in THP-1-derived macrophages. (**a**) Total cellular cholesterol measured in THP-1 macrophages. (**b**) Extracellular HDL levels measured in culture supernatants. The data relating to each condition is expressed as mean ± SD, with *p ≤ 0.05 and **p ≤ 0.01 indicating statistically significant differences.

To validate the role of ApoA1–ABCA1 interaction in cholesterol efflux, total cellular cholesterol in macrophages was quantified by HPLC analysis. After 24 h of ApoA1 stimulation, macrophages incubated with ApoA1 showed a significant decrease in cholesterol content (6.48 ± 0.16 mg/dL, *p* ≤ *0.01*), versus untreated controls (9.25 ± 0.21 mg/dL; Fig. 3a). This significant reduction supports the hypothesis that ApoA1 facilitates cholesterol efflux, likely through its interaction with ABCA1 transporter.

### ApoA1 induces extracellular HDL levels

ApoA1, as the principal structural and functional component of HDL, facilitates cellular cholesterol efflux through direct interaction with ABCA1 transporters at the plasma membrane, thereby initiating nascent HDL particle formation. To corroborate this mechanism, we quantified extracellular HDL levels in macrophage culture supernatants. At 24 h post-stimulation of ApoA1, ApoA1-treated cells exhibited a significant increase in extracellular HDL (2.64 ± 0.15 mg/dL, *p* ≤ 0.05), compared to untreated controls (1.61 ± 0.08 mg/dL; Fig. 3b). This marked elevation in extracellular HDL underscores ApoA1-mediated cholesterol efflux driven by its functional association with ABCA1.

### ApoA1-primed MΦ resists *Ld*-infection

**Fig. 4:**
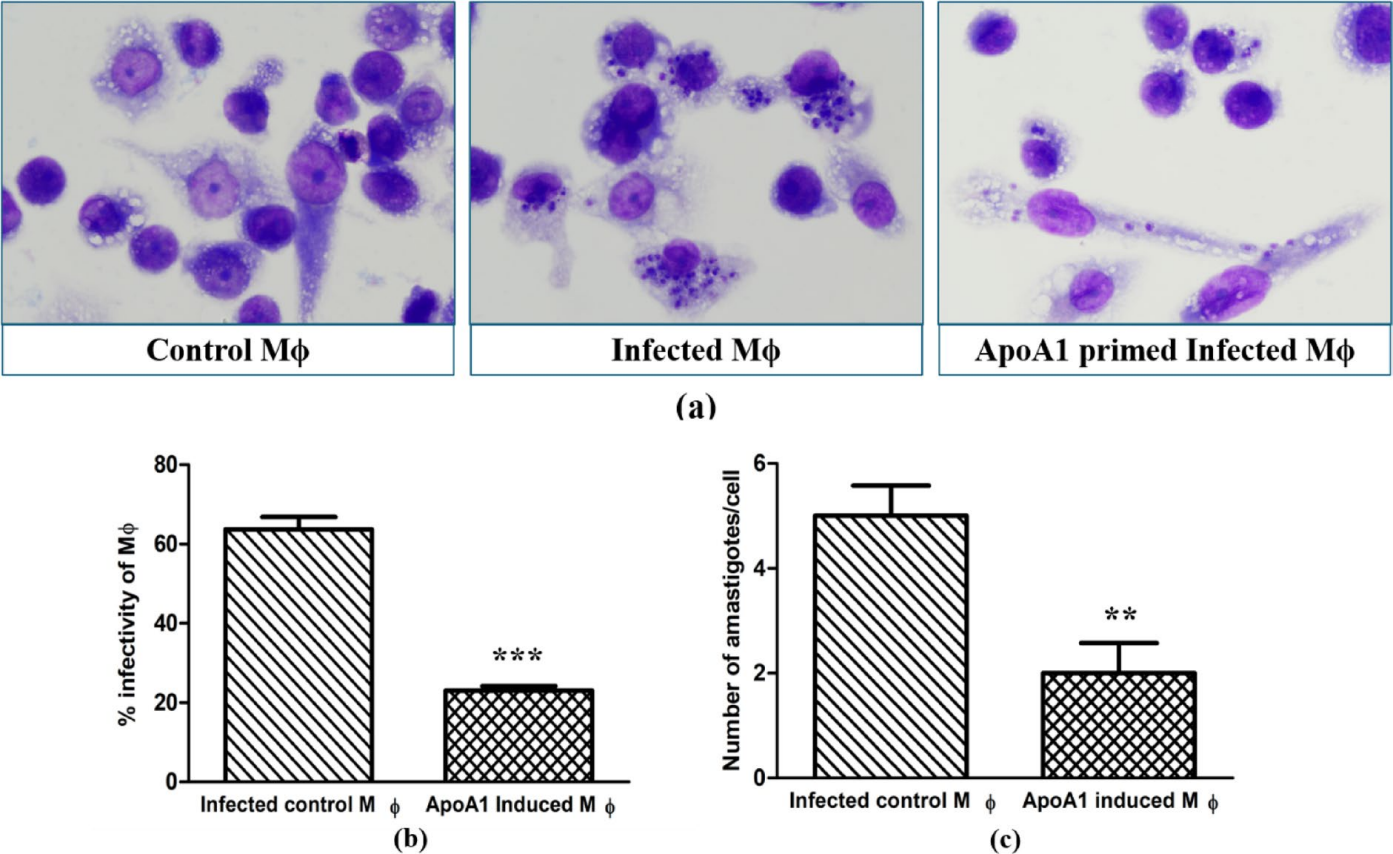
ApoA1 effect on *Ld*-infectivity in THP-1 macrophages. (**a**) Representative light microscopy images of Giemsa-stained MΦ show the absence or presence of intracellular amastigotes in different experimental groups. (**b**, **c**) Quantitative bar graphs illustrate the percentage of infected macrophages and mean intracellular amastigote burden per macrophage, respectively, in infected control and ApoA1-induced Mϕ. The data relating to each condition is presented as mean ± SD, with **p ≤ 0.01 and ***p ≤ 0.001 indicating statistically significant differences.

Cellular cholesterol plays a vital role within the plasma membrane in orchestrating lipid rafts and serves as a crucial energy source, facilitating the attachment, internalization, and subsequent survival of *Leishmania* within MΦ. ApoA1-induced cholesterol efflux from MΦ may disrupt this process. To investigate this hypothesis, we assessed parasite infectivity following ApoA1 priming. Notably, we observed a marked reduction in infected MΦ percentage, 23 ± 2 (*p* ≤ *0.001*), in the ApoA1-treated group versus 63.66 ± 5.5 in the untreated group, representing an approximately 2.78-fold decrease (Fig. 4b). Furthermore, the average number of amastigotes per infected cell dropped from 5 ± 1 in control cells to 2 ± 1 (*p* ≤ *0.01*) in ApoA1-primed MΦ (Fig. 4c). These findings highlight the potential of ApoA1-mediated cholesterol efflux to impair *Leishmania* infectivity and intracellular survival within host MΦ.

### ApoA1 downregulates M2 macrophage signaling

**Fig. 5:**
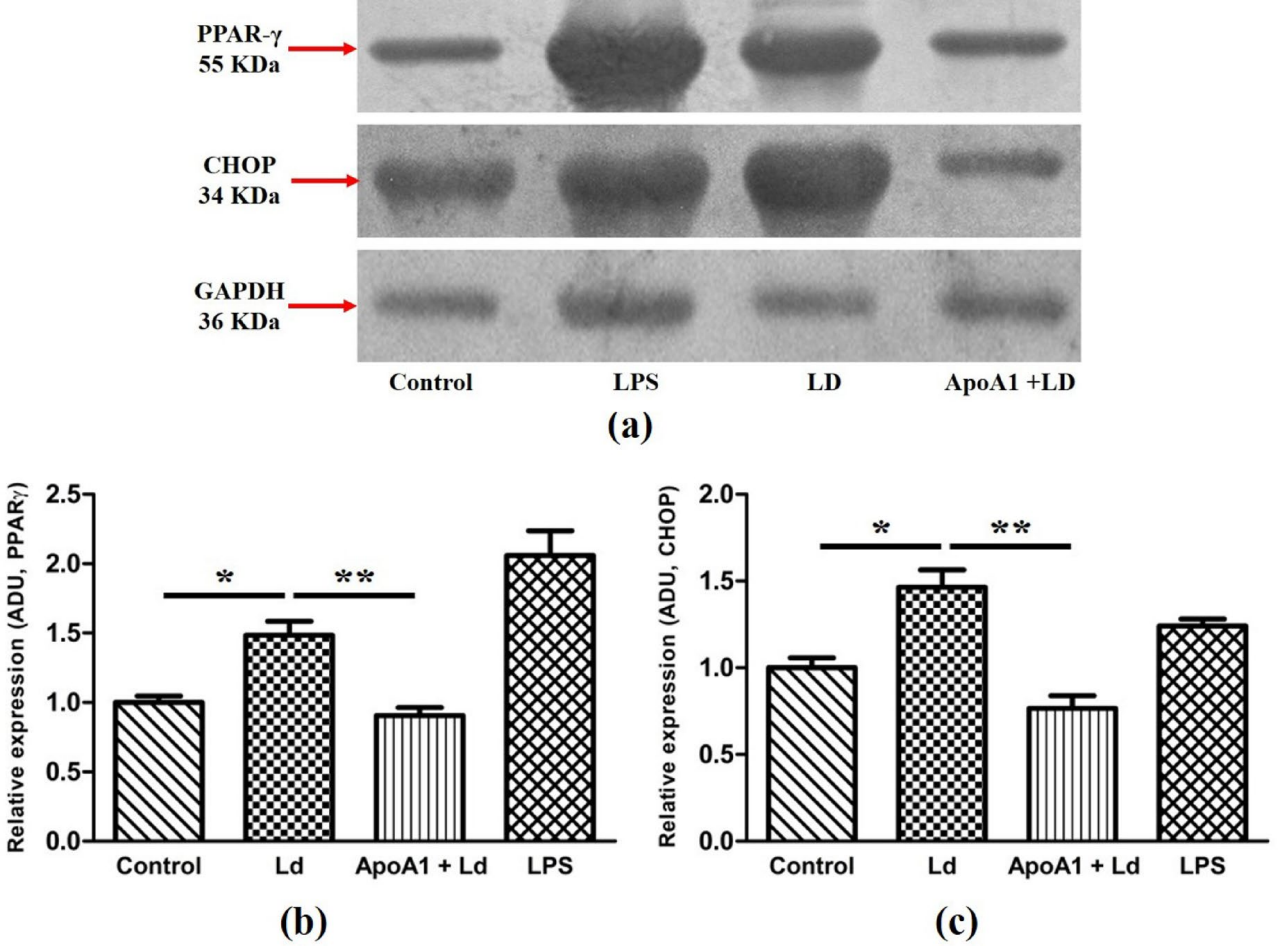
ApoA1 effect on M2 macrophage signaling molecules expression. (**a**) Immunoblotting of CHOP and PPARγ protein expression in macrophages of different experimental groups, and GAPDH was an internal loading control. (**b**, **c**) Bar graphs show the relative density (ADU) of CHOP and PPARγ expression upon normalization with GAPDH. Data are presented as mean ± SD, with *p ≤ 0.05 and **p ≤ 0.01 indicating statistically significant differences.

To further confirm ApoA1-mediated M1 polarization of MΦ, the study evaluated the downregulation of signaling molecules associated with M2 polarization, specifically CHOP and PPAR-γ. In comparison to the uninfected control MΦ, the Ld-infected MΦ represented a 1.46-fold elevation in CHOP expression. In contrast, in ApoA1-primed and Ld-infected MΦ, CHOP expression was reduced by ~ 1.92-fold relative to the Ld-infected group. Similarly, PPAR-γ levels were elevated by ~ 1.48-fold after infection relative to the uninfected control, whereas ApoA1 priming followed by *Ld* infection led to a ~ 1.64-fold decrease in PPAR-γ expression compared to the Ld-infected group (Fig. 5), suggesting the role of ApoA1 in blocking M2 polarization during *Leishmania* infection. LPS stimulation served as a positive control for M2 signaling downregulation, and densitometric analysis was normalized with GAPDH expression.

### ApoA1 induces proinflammatory cytokine response: a hallmark of M1-polarization

**Fig. 6:**
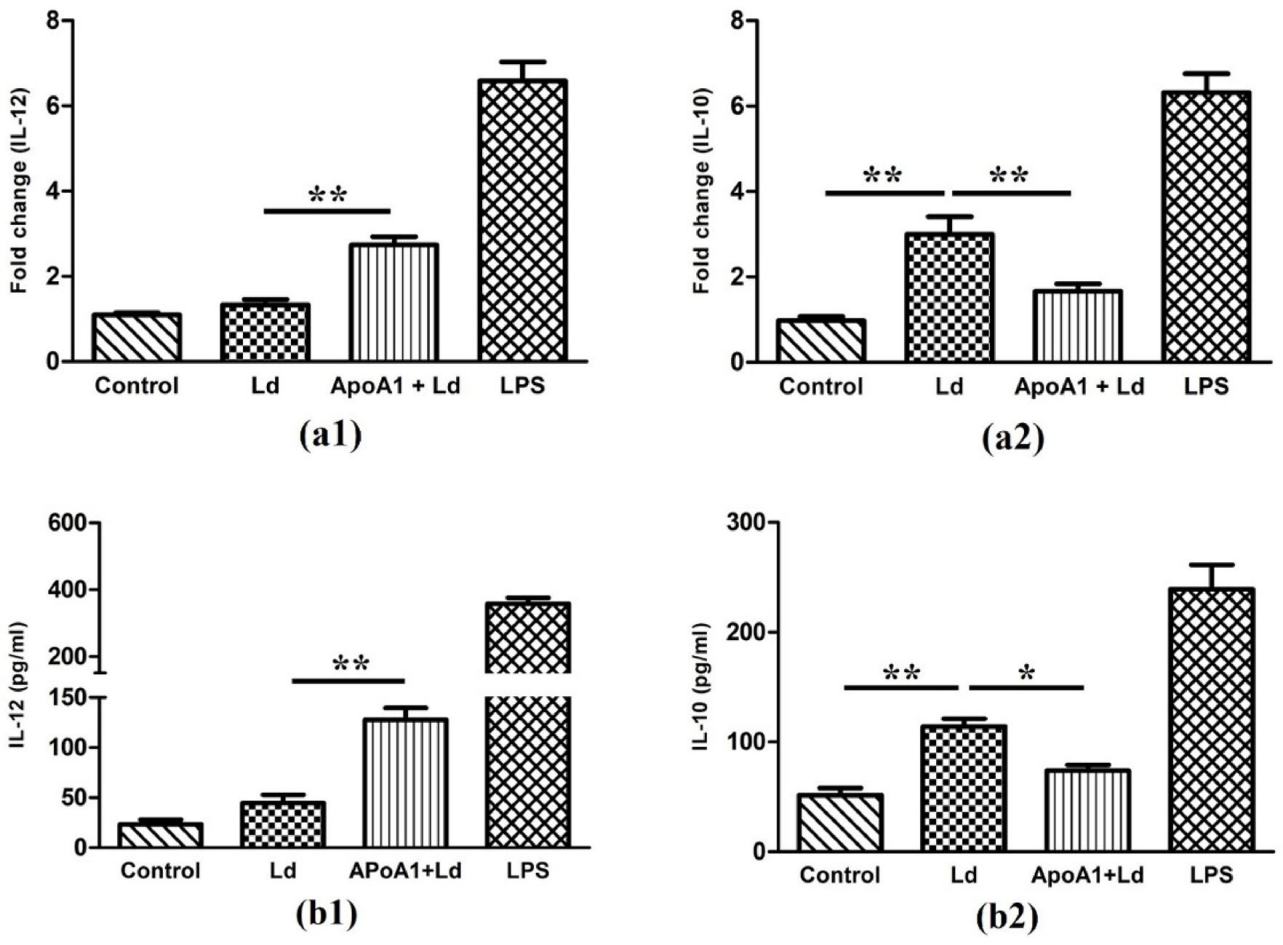
ApoA1 effect on M1 macrophage-specific cytokine response. The bar graphs depict IL-12 and IL-10 levels as (**a**) fold change in mRNA expression, and (**b**) protein concentration (pg/ml) in culture supernatant, in different experimental groups. Data are presented as mean ± SD, with *p ≤ 0.05 and **p ≤ 0.01 indicating statistically significant differences.

To define the role of ApoA1 in modulating MΦ polarization, we assessed pro- and anti-inflammatory mediators expression profiles at both the transcriptional as well as translational levels. Using quantitative PCR, we observed that control MΦ maintained baseline expression of IL-12 and IL-10. Upon Ld-infection, IL-12 mRNA expression remained unaltered, whereas IL-10 mRNA was markedly upregulated by 2.99-fold (*p* ≤ 0.01). In contrast, ApoA1-primed and Ld-infected MΦ exhibited a robust induction of IL-12 expression (2.73-fold; *p* ≤ 0.01), and downregulation in IL-10 (1.66-fold; *p* ≤ 0.01) (Fig. 6a). In correlation with the mRNA results, ELISA-based quantification revealed that control MΦ secreted baseline levels of IL-12 (23.36 ± 7.83 pg/mL) and IL-10 (51.71 ± 10.89 pg/mL). The Ld-infection results in increased secretion of IL-12 (44.53 ± 14.43 pg/mL; *p* ≤ 0.05) and IL-10 (114.10 ± 12.11 pg/mL; *p* ≤ 0.01). However, in ApoA1-primed and Ld-infected MΦ, IL-12 production increased (127.67 ± 20.59 pg/mL; *p* ≤ 0.01) and IL-10 levels decreased (73.88 ± 9.16 pg/mL; *p* ≤ 0.05) significantly compared to Ld-infected MΦ (Fig. 6b). These results highlight the role of ApoA1 in M1 macrophage-driven cytokine response against *Leishmania* attack.

### ApoA1 regulates (inducible nitric oxide synthase) iNOS and Arginase-1 expression in favour of the host

**Fig. 7:**
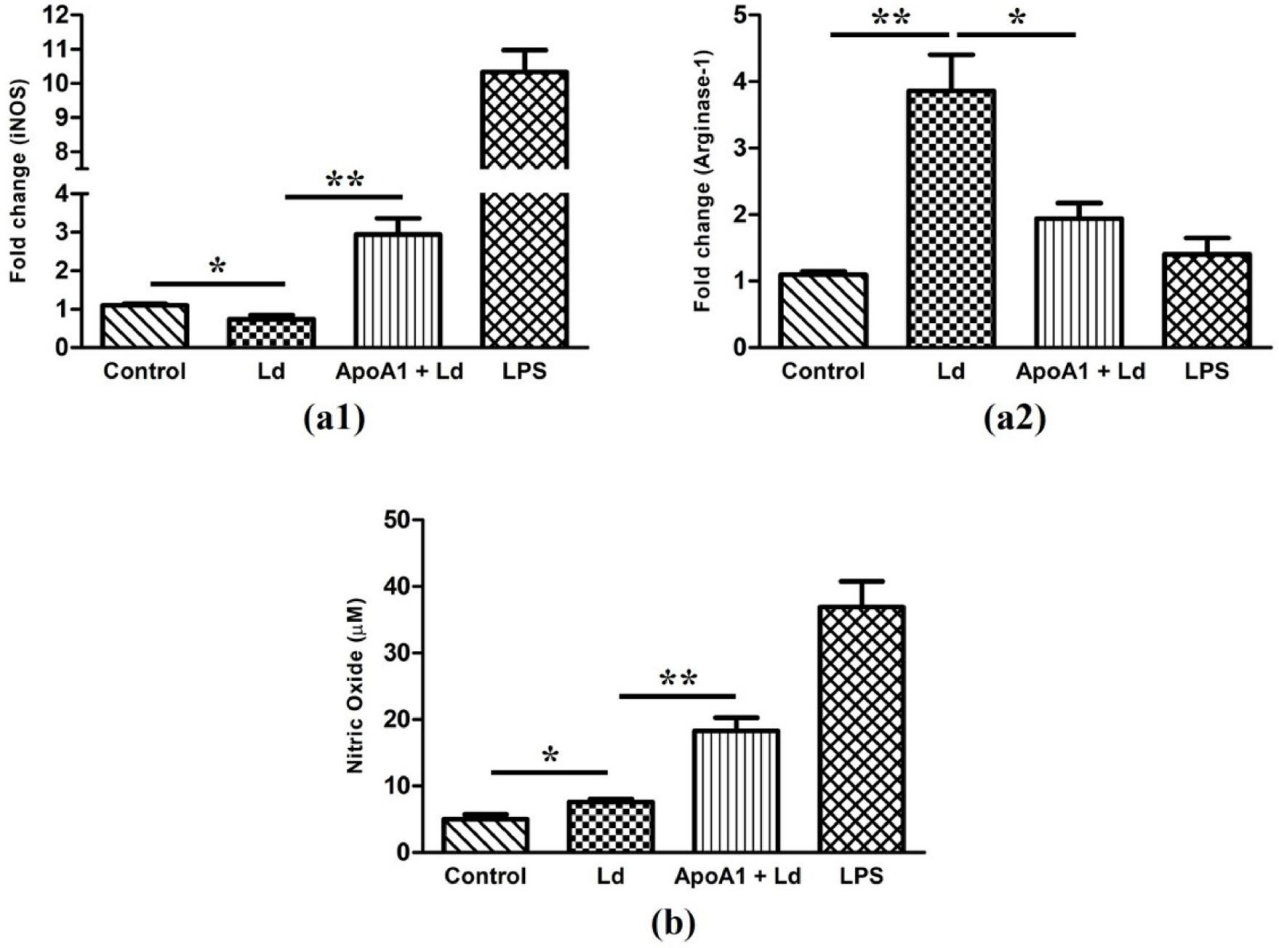
ApoA1 effect on key determinants of macrophage during *Ld*-infection. The bar graphs show (**a**) fold change in mRNA expression of iNOS and Arginase-1, and (**b**) nitric oxide production (µM), in different experimental groups. The data relating to each condition is presented as mean ± SD, with *p ≤ 0.05, and **p ≤ 0.01 indicating statistically significant differences.

As a final validation of M1-polarization, we measured iNOS2 (M1marker) and Arginase-1 (M2 marker) gene expression in MΦ. In control, both the markers were expressed at basal levels. Upon *Ld*-Infection, iNOS2 expression was diminished by 1.56-fold (*p* ≤ 0.01), and Arginase-1 expression was upregulated by 3.86-fold (*p* ≤ 0.01). Conversely, in ApoA1-primed and *Ld*-infected MΦ, iNOS2 expression was upregulated by 2.93-fold (*p* ≤ 0.01) and Arginase-1 expression was downregulated by 2-fold (*p* ≤ 0.05) compared to the *Ld*-infected group (Fig. 7a). In correlation to the induced iNOS2 expression, the NO levels were also found to be upregulated in ApoA1-primed and *Ld*-infected MΦ (18.31 ± 1.40 µM; *p* ≤ 0.001) compared to the *Ld*-infected MΦ (6.60 ± 1.26 µM), reinforcing the antimicrobial potential of ApoA1 via induction of M1 polarization. In control MΦ, the NO levels (5 ± 1.22 µM) were at baseline and did not alter much (*p* ≤ 0.05) upon *Ld-*infection. LPS was used as a positive control (36.88 ± 6.71 µM) (Fig. 7b).

### ApoA1-primed MΦ co-culture upregulates Th1-specific transcription factors

**Fig. 8:**
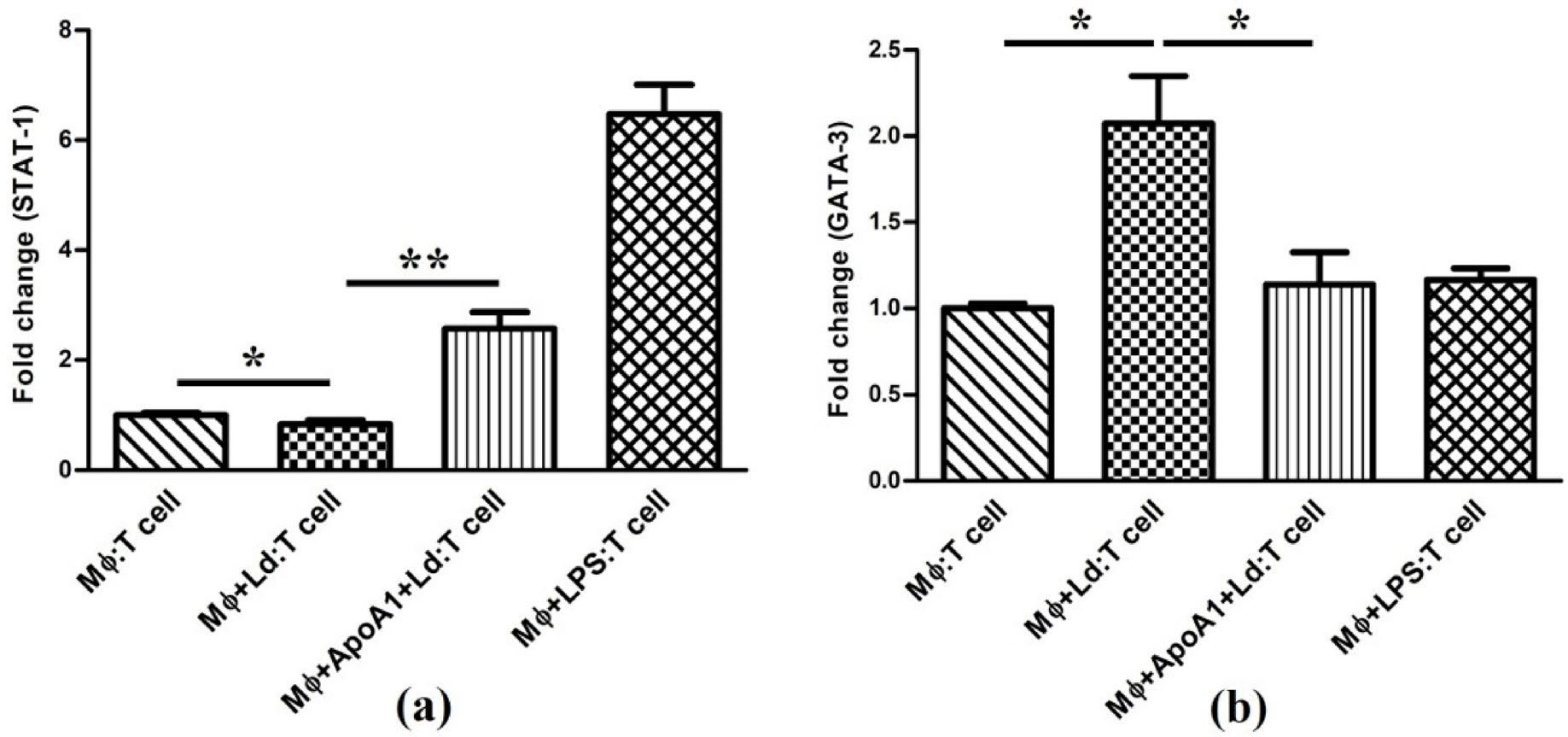
ApoA1 effect on T-cell lineage-specific transcription factors expression in Mϕ: T cell co-culture experiments. The presented bar graph illustrate the relative fold induction of (**a**) STAT-1 and (**b**) GATA-3 transcripts in distinct experimental conditions: Control (Mϕ: T cell), Infected Mϕ: T cell (Mϕ + Ld: T cell), ApoA1 primed infected Mϕ: T cell (Mϕ + ApoA1 + Ld: T cell), and LPS induced Mϕ: T cell (Mϕ + LPS: T cell). The data relating to each condition is presented as mean ± SD, with *p ≤ 0.05 and **p ≤ 0.01 indicating statistically significant differences.

The PBMC-derived T-cell phenotype was confirmed by Flow Cytometry analysis, and observed that > 80% cells were positive for CD3 expression. For further assessment of ApoA1-primed MΦ influence on T-cell differentiation, the MΦ—naïve T-cell co-culture system was employed. The expression of lineage-specific transcription factors, e.g., STAT-1 (Th1) and GATA-3 (Th2), along with their associated cytokines, IFN-γ and IL-4, was examined to define the Th1/Th2 polarization. In *Ld*-infected MΦ co-culture, the expression of STAT-1 was slightly reduced (*p* > 0.05) relative to the control. However, in ApoA1-primed and *Ld*-infected MΦ co-culture, it was robustly upregulated (3.21-fold; *p* < 0.01) and indicating a strong pro-Th1 skewing effect. Conversely, GATA-3, the central regulator of Th2 polarization, was significantly upregulated (2.07-fold; *p* < 0.05) in T-cells co-cultured with *Ld*-infected MΦ relative to the control. Whereas, in ApoA1-primed and *Ld*-infected MΦ co-culture, it was significantly downregulated in T-cells (1.83-fold; *p* < 0.05) compared to the *Ld*-infected MΦ co-culture, indicating the role of ApoA1 in suppression of *Leishmania*-driven Th2-skewing (Fig. 8).

### ApoA1-primed MΦ co-culture triggers Th1-type cytokine response

**Fig. 9:**
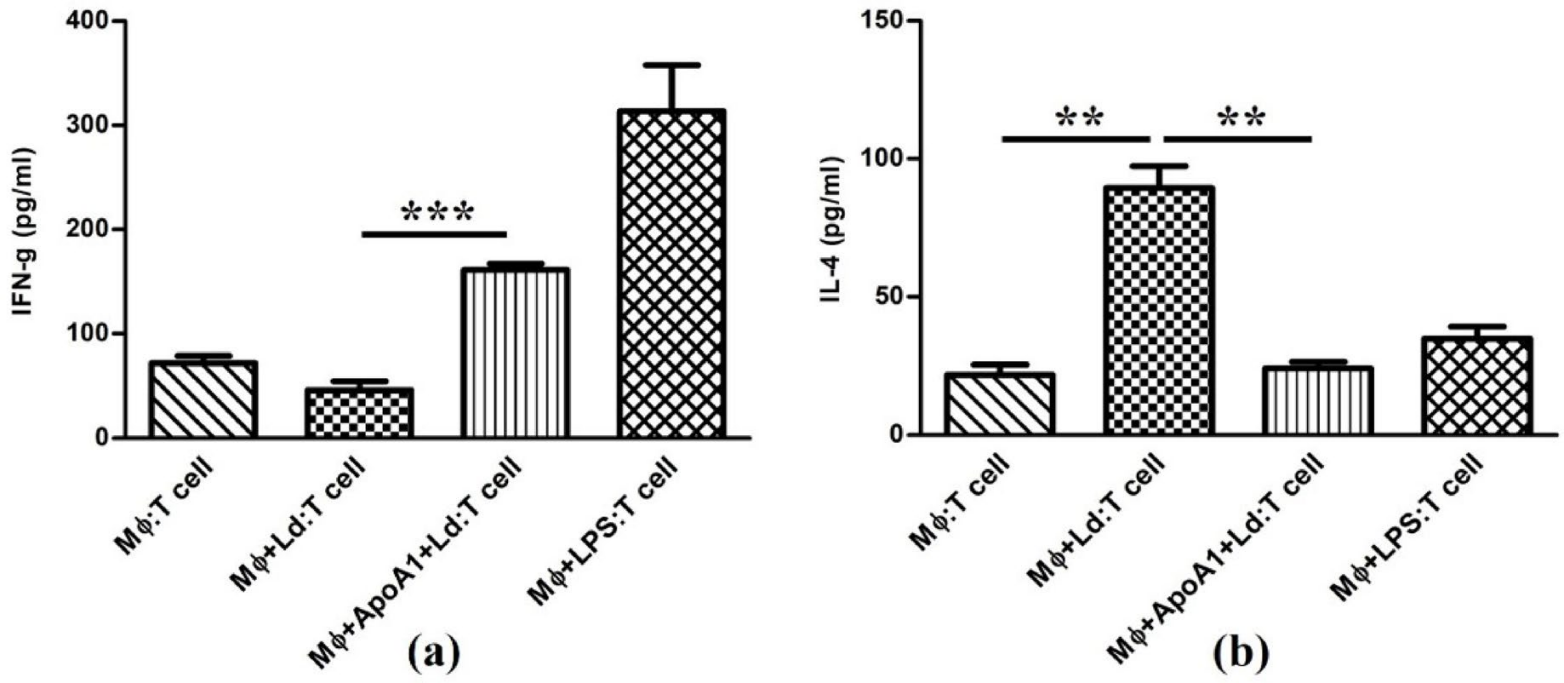
ApoA1 effect on Th1/Th2 cytokine production in Mϕ: T cell co-culture experiments. The bar graphs show levels of (**a**) IFN-γ (Th1) and (**b**) IL-4 (Th2) in culture supernatants of different experimental groups. Control (Mϕ: T cell), Infected Mϕ: T cell (Mϕ + Ld: T cell), ApoA1 primed infected Mϕ: T cell (Mϕ + ApoA1 + Ld: T cell), and LPS induced Mϕ: T cell (Mϕ + LPS: T cell). The data relating to each condition is represented as mean ± SD, with **p ≤ 0.01 and ***p ≤ 0.001 indicating statistically significant differences.

To validate the transcriptional data at the functional level, secretion of Th1-linked IFN-γ and Th2-linked IL-4 was quantified by ELISA. In *Ld*-infected MΦ co-culture, the IFN-γ production was lower (46.32 ± 14.76 pg/mL; *p* ≥ 0.05) than control (72.24 ± 11.69 pg/mL). Whereas, in ApoA1-primed and *Ld*-infected MΦ co-culture, the IFN-γ production was significantly higher (161.22 ± 10.39 pg/mL; *p* ≤ 0.001) than in *Ld*-infected MΦ co-culture, reinforcing the translational evidence of Th1 polarization by ApoA1. On the contrary, IL-4 levels were substantially upregulated in the *Ld*-infected MΦ co-culture (89.45 ± 13.54 pg/mL; *p* ≤ 0.01) relative to the control (21.83 ± 6.41 pg/mL). However, in ApoA1-primed and *Ld*-infected MΦ co-culture, IL-4 production was substantially declined (24.21 ± 4.06 pg/mL; *p* ≤ 0.01) compared to *Ld*-infected MΦ co-culture, demonstrating the translational evidence of Th2 suppression by ApoA1 (Fig. 9).

## Discussion

Lipids are fundamental biomolecules required for the survival and pathogenesis of *Leishmania* within mammalian MΦ and serve as critical sources for both energy production and membrane biogenesis^24,25^. During the promastigote stage, *Leishmania* primarily depends on de novo lipid biosynthesis, producing key lipid classes such as glycerophospholipids, sterols, and sphingolipids. However, in the intracellular amastigote stage, the parasite undergoes a metabolic shift and predominantly acquires lipids directly from the host MΦ^26^, highlighting the significance of host lipid metabolism in supporting parasite persistence. ApoA1, the major structural protein in HDL, is integral to the regulation of cellular lipid homeostasis. Previous studies have reported that patients with VL exhibit an impaired lipid profile, characterized by distinctly reduced levels of ApoA1 and HDL^8,9,27^. Consistent with these findings, our data also demonstrate a marked reduction in serum ApoA1 levels in VL patients, supporting the link between impaired lipid metabolism and disease pathophysiology.

In spite of this evidence, the functional implications of low serum ApoA1 levels during *Leishmania* infection have still been largely unexplored. Specifically, whether low ApoA1 availability contributes to parasite survival by modulating host lipid dynamics has not been previously addressed. To bridge this gap, our study is concentrated firstly on examining the immunomodulatory role of ApoA1 during *Leishmania* infection, with a particular emphasis on its interaction with the ABCA1 transporter and its subsequent effects on MΦ cholesterol management. Furthermore, ApoA1 is well known for cholesterol and phospholipid efflux via interaction with the ABCA1 transporter, particularly from MΦ^28^. Using confocal microscopy, we confirmed the co-localization of ApoA1 with ABCA1, indicating a physical interaction on MΦ plasma membrane. Additionally, we have found that ApoA1 stimulation resulted in a notable reduction in total cellular cholesterol, aligning with its role in promoting cholesterol efflux. Furthermore, we noticed that ApoA1 stimulation not only promotes cholesterol efflux but also is involved in extracellular HDL formation, as evidenced by the significant upsurge in HDL levels subsequent to ApoA1 stimulation.

The regulation of cholesterol dynamics is crucial in maintaining the integrity of membrane lipid rafts in MΦ, which are important for modulating host-parasite interactions, enabling parasite internalization, forming the parasitophorous vacuole, and activating immune signaling pathways^29–31^. Inclusively, these processes significantly impact the internalization and survival of parasites within host cells. In this study, we provide convincing evidence that ApoA1 stimulation markedly reduces *Leishmania* infectivity, as indicated by a pronounced reduction in both the proportion of infected MΦ and the intracellular amastigote burden. This reduction in infectivity is ascribed to ApoA1–ABCA1-mediated cholesterol efflux, which diminishes membrane cholesterol and disrupts lipid raft microdomains critical for parasite entry. Furthermore, enhanced cholesterol efflux likely restrains the intracellular cholesterol pool, which is essential for the biosynthesis and maintenance of the parasitophorous vacuole, thereby compromising the intracellular niche required for survival and multiplication of *Leishmania* within MΦ. Collectively, these results suggest that ApoA1-mediated regulation of macrophage cholesterol dynamics is a crucial determining factor of cellular susceptibility to *Leishmania* infection.

MΦ function as the principal host cells and central immunological regulators in determining the fate of *Leishmania* parasites, playing a decisive role in either supporting parasite clearance or facilitating intracellular survival^32^. The infection outcome is largely governed by the polarization state of the MΦ, which can exist along a spectrum between the M1 and M2 states^5,32^. M1, or classically activated MΦ, are defined by secretion of cytokines like IL-12 & TNF-α, elevated ROS (reactive oxygen species) and NO production via iNOS, and surface expression of CD86, all of which contribute to effective parasite killing. In contrast, M2 macrophages, or alternatively activated MΦ, promote parasite persistence by producing immunosuppressive cytokines like IL-10 & TGF-β, and by expressing CD163, CD206, and arginase-1, which support tissue repair and immune suppression^33,34^. The ApoA1-mediated cholesterol efflux is associated with M2 polarization of MΦ, which is responsible for anti-inflammatory response^26^, which may be associated with parasite succession. Despite prior evidence highlighting the anti-inflammatory roles of HDL and ApoA1^35–40^, the recent investigations are focused on unravelling their role in pro-inflammation as well^40–43^. In substantiation of this statement, our study demonstrated ApoA1-mediated extracellular HDL formation and its influence on proinflammatory response, possibly mediated by M1 macrophages. This is evidenced by increased expression of IL-12 at both transcriptional and translational levels, upregulation of iNOS, and elevated NO production. Collectively, these findings indicate that ApoA1 drives polarization of infected MΦ toward a pro-inflammatory M1 phenotype. CHOP and PPARγ are critical transcriptional regulators associated with the M2 macrophage phenotype, which promotes the anti-inflammatory response and a conducive environment for *Leishmania* survival^43–47^. In our study, *Ld-*infection alone in MΦ shows elevated expression of both CHOP and PPAR-γ, whereas ApoA1-priming of these MΦ significantly inhibited their expression, suggesting ApoA1’s modulatory effect in the macrophage activation towards the inhibition of M2 polarization during *Leishmania* infection.

Macrophages are key regulators of adaptive immunity through the modulation of naïve T lymphocyte differentiation^5^. The phenotype of macrophages can influence the development of Th cell subsets, directing the immune polarization toward protective Th1 or pathogenic Th2 responses. Th1 cells, defined by the activation of transcription factors STAT-1& T-bet, leading to production of IFN-γ & TNF-β pro-inflammatory cytokines, which are critical for MΦ activation and effective control of intracellular pathogens such as *Leishmania*. In contrast, Th2 cells, which express GATA3, secrete IL-4 &IL-10, anti-inflammatory cytokines, which can suppress protective immune responses and thereby promote parasite persistence^6^. Functionally, M1 macrophages support Th1-type immune responses, while M2 macrophages favor Th2-type polarization^5^. In the current study, we provide evidence that ApoA1-primed MΦ modulate T cell polarization toward a Th1 phenotype. Co-culture of ApoA1-stimulated, *Ld*-infected macrophages with naïve CD3⁺ T cells resulted in elevated IFN-γ production and STAT-1 expression, indicative of Th1 differentiation. These findings suggest that ApoA1 promotes M1 macrophage polarization, which in turn enhances the Th1-skewing, a process critical for the activation of cell-mediated immunity and effective clearance of *Leishmania* parasites. This highlights a novel immunoregulatory role of ApoA1 in orchestrating innate and adaptive immunity during *Leishmania* infection.

## Conclusion

Overall, our study demonstrates that ApoA1 has a key immunoregulatory potential that can drive MΦ towards an M1 phenotype during *Leishmania* infection, which induces pro-inflammatory response in the vicinity of HDL and drives Th1-skewing to protect the host from VL. These findings highlight ApoA1 as a promising therapeutic modulator capable of reshaping the MΦ microenvironment to favour anti-leishmanial immunity.

## Supplementary Information

Below is the link to the electronic supplementary material.

Supplementary Material 1

## Data Availability

All data generated or analysed during this study are included in this published article [and its supplementary information files].
