# Supplementary material for: ApoA1-driven cholesterol efflux and macrophage polarization orchestrate T-cell differentiation towards controlling Leishmania donovani pathogenesis
Source: Sci Rep. 2025 Dec 3;16:586. doi: 10.1038/s41598-025-30130-1 (PMC12775519; doi:10.1038/s41598-025-30130-1)
Supplement: Supplementary file 1 — Supplementary Material 1 [file 41598_2025_30130_MOESM1_ESM.docx]

**Supplementary materials**

**Table S1: This table represents the raw data of Figure 1.**Table 1 represents the ApoA1 concentration (mg/dl) in three experimental groups: Healthy, VL and PKDH.

| Healthy | VL | PKDL |
| --- | --- | --- |
| 1.609921 | 0.5659918 | 1.492734 |
| 1.509793 | 0.7553082 | 1.335310 |
| 1.852639 | 0.5645084 | 1.519620 |
| 2.435422 | 0.8077828 | 1.371838 |
| 2.219961 | 0.396701 | 1.192164 |
| 1.713758 | 0.8628534 | 1.664621 |
| 1.515170 | 0.5244571 | 1.551884 |
| 1.535937 | 0.5049878 | 1.500707 |
| 1.530004 | 0.415985 | 1.565976 |
| 1.888240 | 0.3601728 | 2.233311 |
| 1.668514 | 0.5849049 | 1.632542 |
| 1.498853 | 0.1195316 | 1.008966 |
| 1.649416 | 0.9754049 | 1.377216 |
| 1.331416 | 0.7022774 | 2.404642 |
| 1.555036 | 0.5455954 | 1.457874 |
| 1.785145 | 0.4510298 | 1.576359 |
| 1.658502 | 0.6049306 | 1.311947 |
| 2.117979 | 0.9479624 | 1.548917 |
| 1.351130 | 0.7180383 | 1.641443 |
| 1.769755 | 0.8033327 | 1.510164 |
| 1.712274 | 0.1259472 | 1.238705 |
| 1.380182 | 0.5697002 | 1.960184 |
| 1.702076 | 0.9145863 | 1.577843 |
| 1.899551 | 0.7069129 | 1.937006 |
| 1.656833 | 0.4139453 | 1.506826 |
| 1.324741 | 0.1068672 | 1.299524 |
| 1.450643 | 0.639790 | 1.576174 |
| 1.877671 | 0.8229875 | 1.692063 |
| 1.343098 | 0.7656919 | 1.682236 |
| 1.683534 | 0.7786715 | 1.473265 |


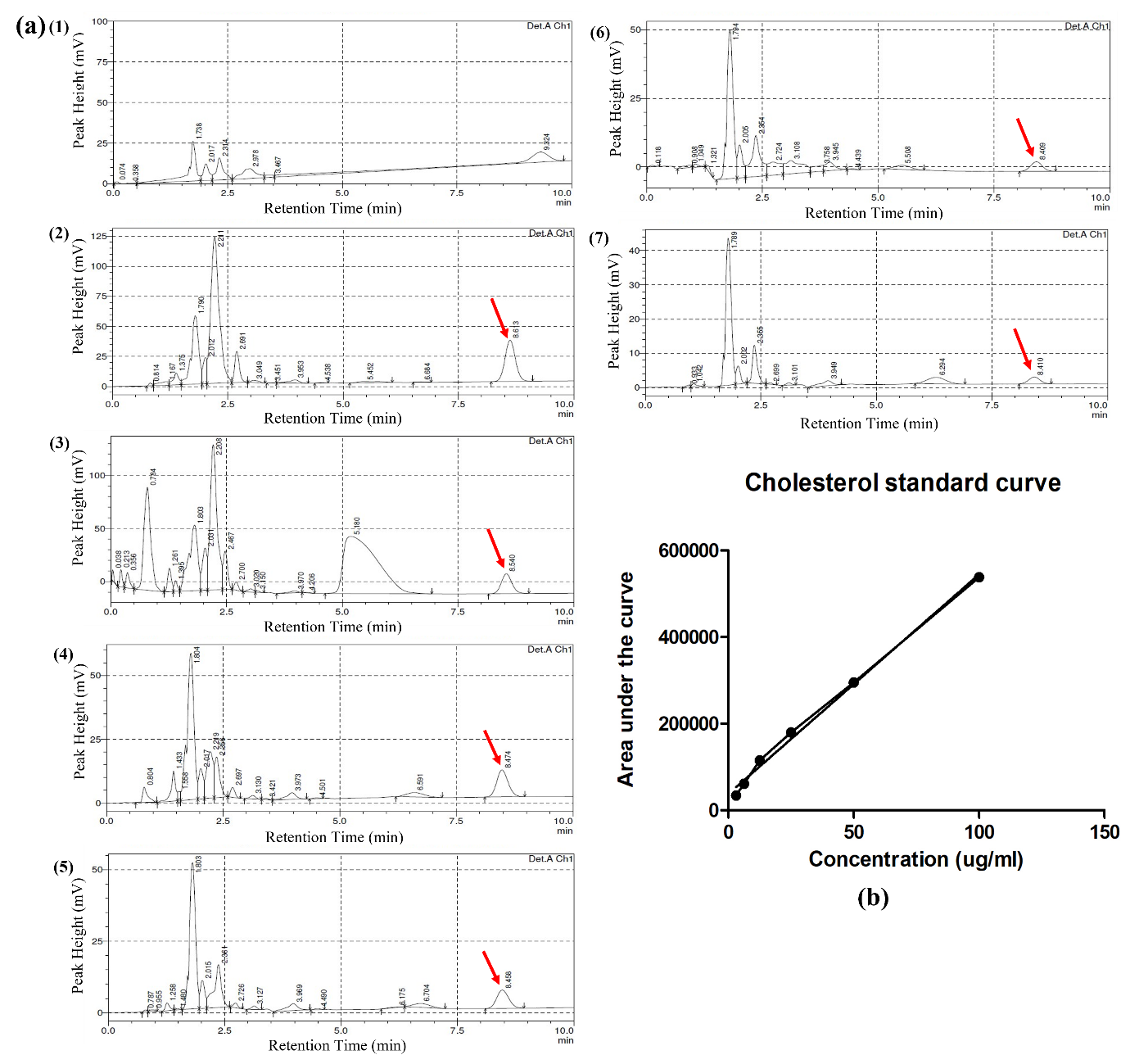


**Figure S1.**Figure a represents the chromatogram of cholesterol standards, including a blank (a1) and a series of concentrations from 100 to 3.125 μg/mL (a2–a7) obtained via 50-fold serial dilutions. Figure b depicts the corresponding standard curve for cholesterol quantification, based on the chromatographic peak areas.


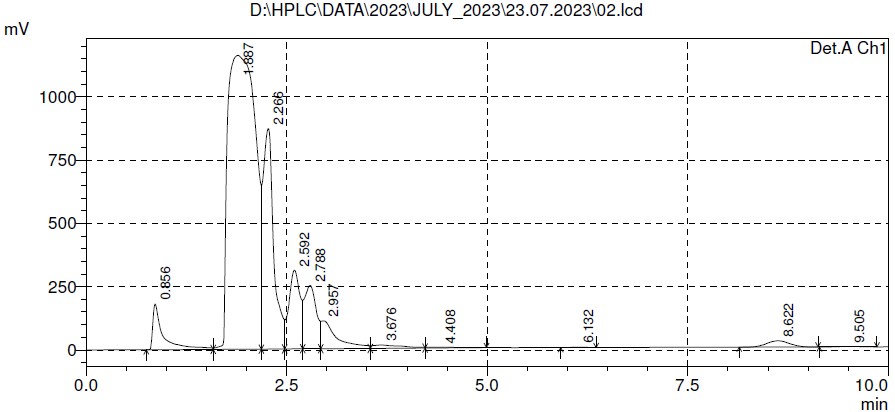


**A**


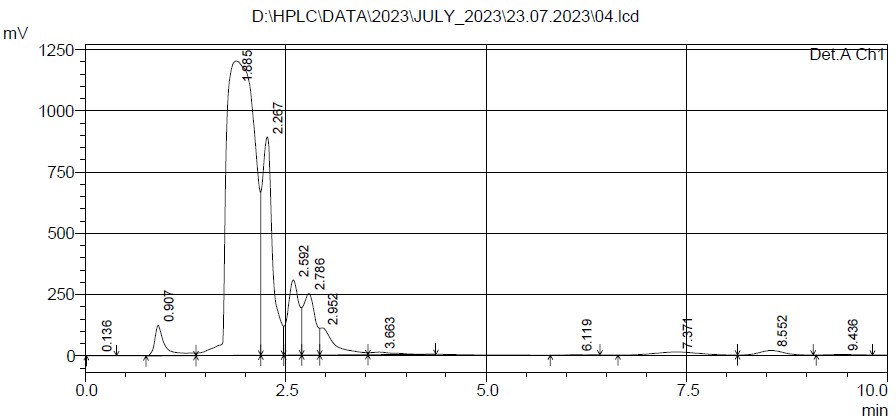


B

**Figure S2.** Figure S2 represents the raw data for **Figure 3a.** Chromatogram of cellular cholesterol. (A) represents the control macrophage, while (B) ApoA1-stimulated macrophage**.**


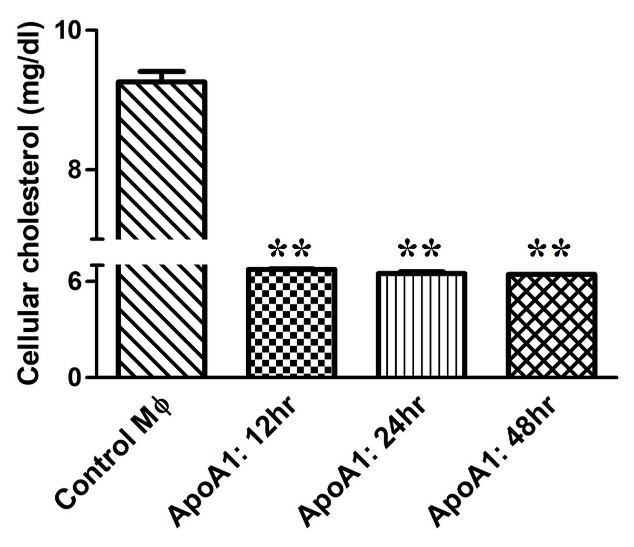


**Figure S3.** Figure S3 represents the cellular cholesterol level (mg/dl) in control macrophage and in ApoA1 (10 µg/mL) stimulated macrophage at 12hr, 24hr, and 48hr.

**Table S2:** This table represents the raw data of Figure 3d.Table S2represents HDL level (mg/dl) in the culture supernatant of control and ApoA1-stimulated macrophage.

| Control Mϕ | ApoA1 stimulated Mϕ |
| --- | --- |
| 1.67 | 2.53 |
| 1.55 | 2.74 |


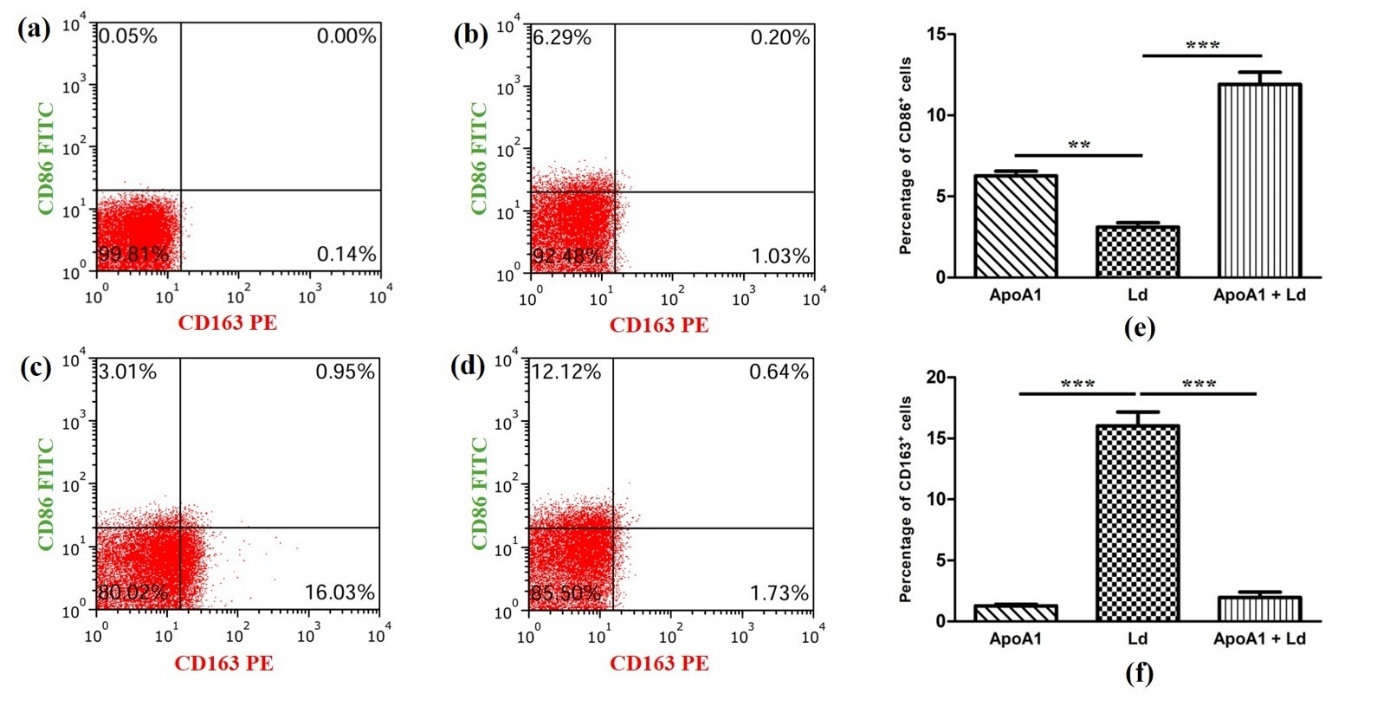


**Figure S4.** Flow cytometric analysis of macrophage cell surface markers CD86 (M1) and CD163 (M2). The dot plot with quadrant gating shows the percentage of naïve and M1/M2 polarized macrophages distribution in different experimental groups: (a) Control Mϕ, (b) ApoA1-primed Mϕ, (c) Ld-infected Mϕ, and (d) ApoA1-primed Ld-infected Mϕ. (e & f) The bar graphs explain the % of of CD86+ & CD163+ cells, respectively, from three independent experiments. The data relating to each condition is presented as mean ± SD, with **p≤0.01 and ***p≤0.001 indicating statistically significant differences.


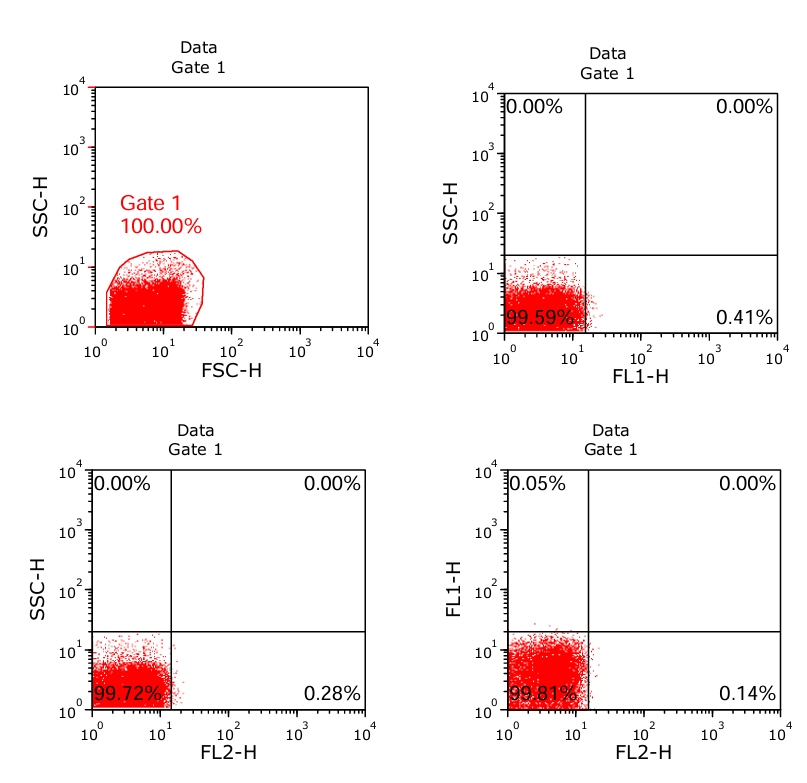
**Raw data for Flow cytometry for CD-86 and CD-163 analysis: a-d**


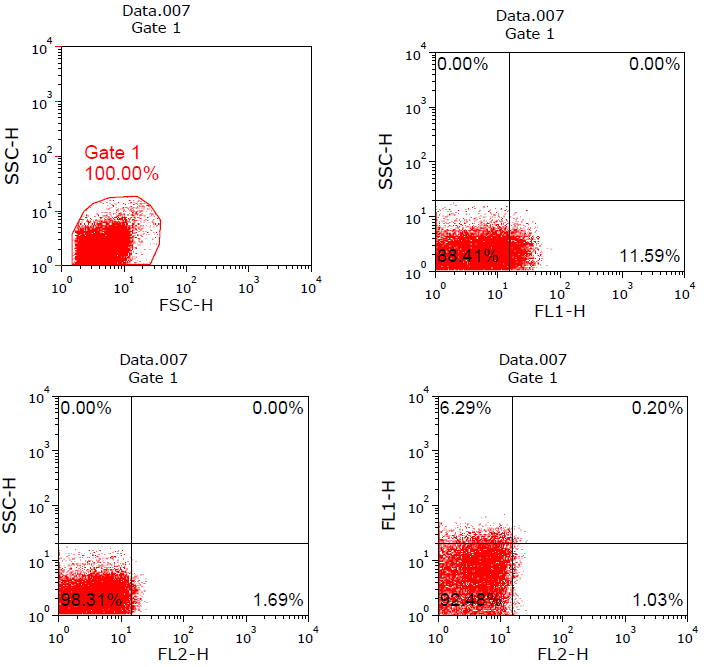
**Figure S5.** Raw data for Figure S4a**.** Dot plot of unstained control macrophage

**Figure S6.** Raw data for Figure S4b. Dot plot of ApoA1-stimulated macrophage


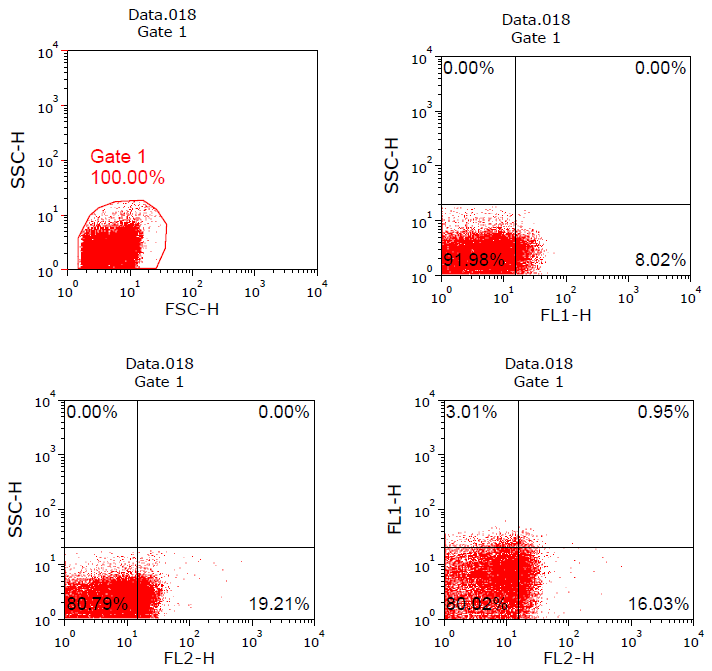


**Figure S7.** Raw data for Figure S4c. Dot plot of *Ld* infected macrophage


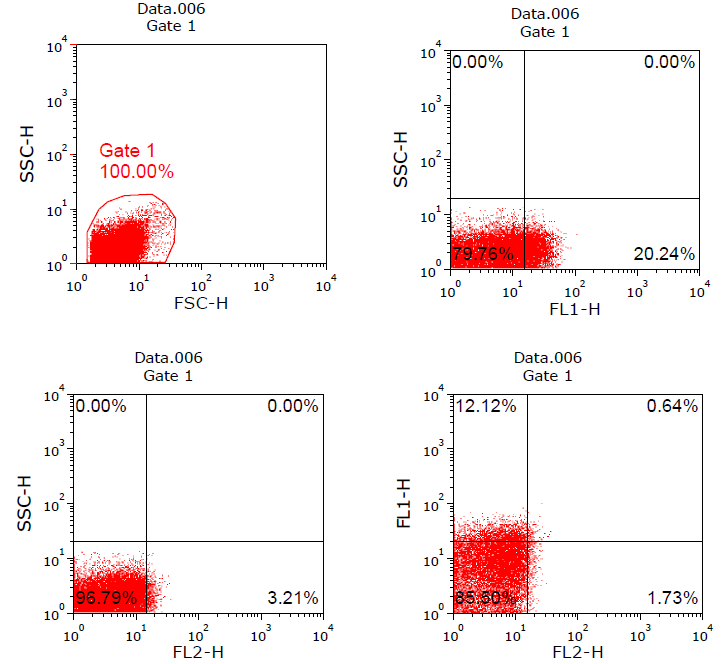


**Figure S8.** Raw data for Figure S4d. Dot plot of ApoA1-stimulated and infected macrophage


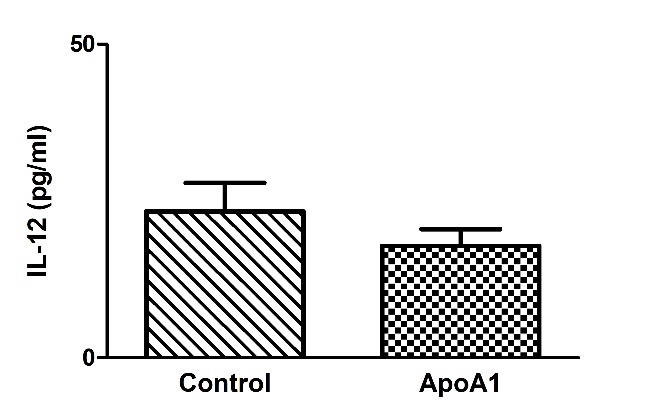

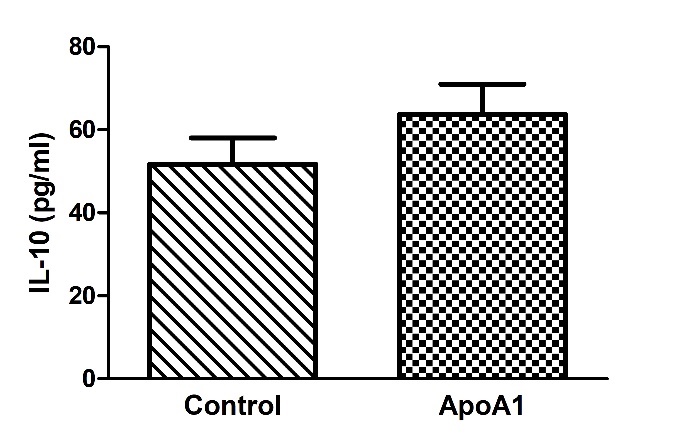


**B**

**A**

**Figure S9.** Figure S8 represents the level of IL-10 (A) and IL-12 (B) in control and ApoA1-only control.

**Raw data of western blot (Figure 5)**


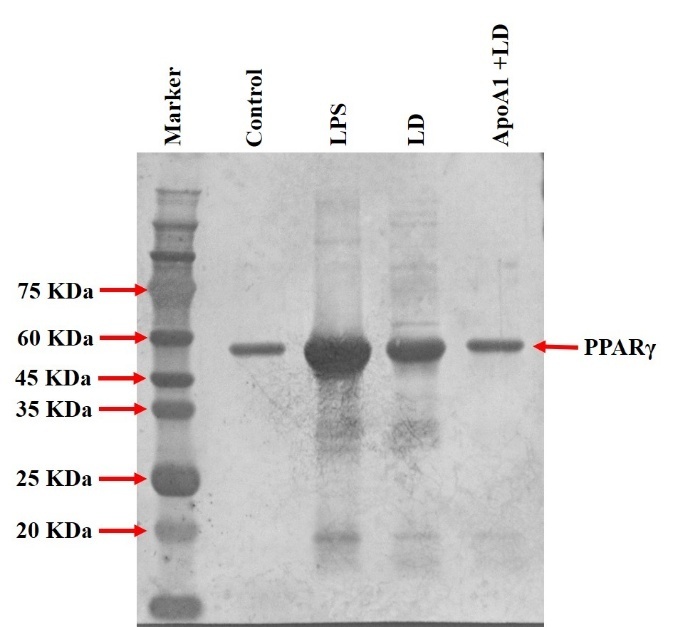

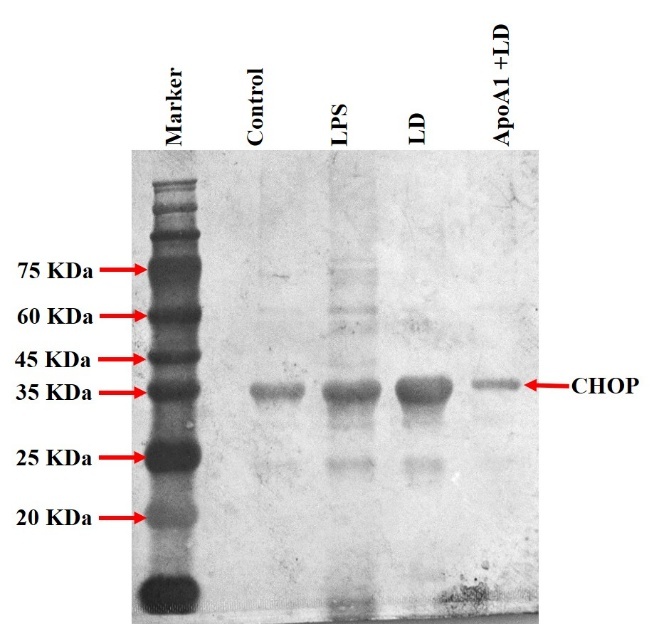


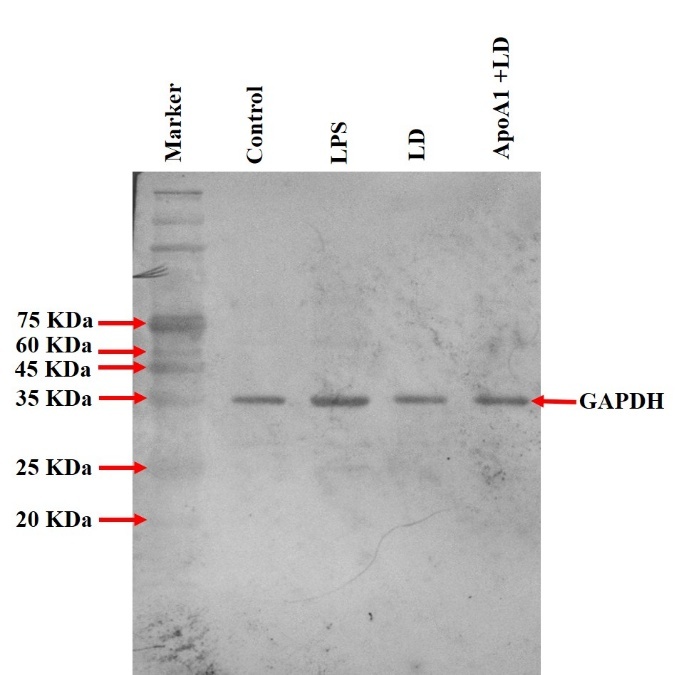


**Figure S11** represents a Blot of CHOP

**Figure S10.**Represents a Blot of PPAR-ϒ

**Figure S12** represents a Blot of GAPDH

**TableS3:** Table showing data of Figure 7b.

| Control | Ld | ApoA1 + Ld | LPS |
| --- | --- | --- | --- |
| 4.9892 | 7.7887 | 21.5586 | 31.0096 |
| 3.7893 | 8.2765 | 18.5894 | 35.4280 |
| 6.2431 | 6.7508 | 14.7833 | 44.2087 |

Table S3 represents to the Nitric Oxide level (µM) in differential conditions: Control macrophage,*Ld*infected macrophage, ApoA1 stimulated infected macrophage, and LPS induced macrophage

**Raw data of T cell characterization using Flow Cytometry**


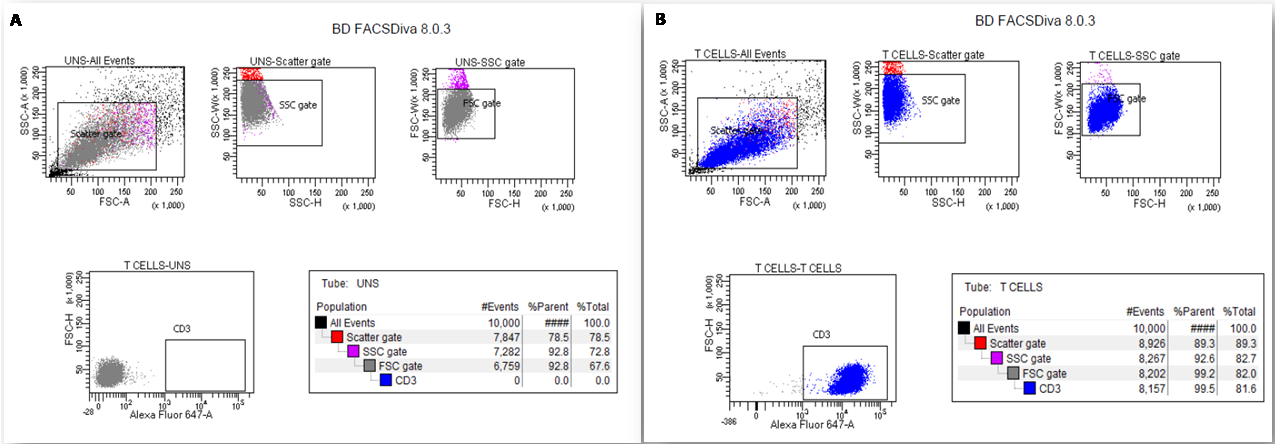


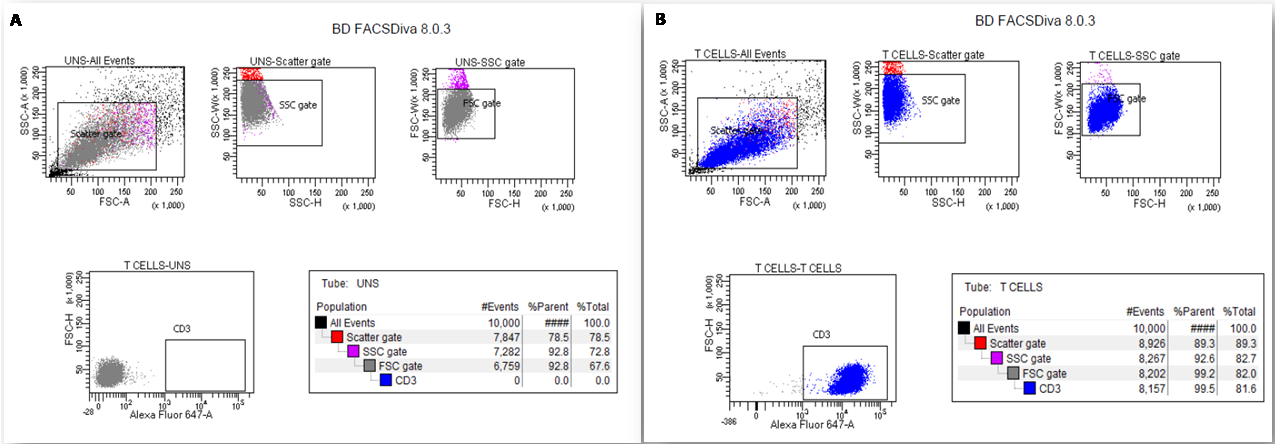


**Figure S13** represents dot plots of Unstained T cells(**A**), and stained T cells(**B**).
